# Single Electron Activation of Aryl Carboxylic Acids

**DOI:** 10.1016/j.isci.2020.101266

**Published:** 2020-06-12

**Authors:** Xiao-Qiang Hu, Zi-Kui Liu, Ye-Xing Hou, Yang Gao

**Affiliations:** 1 Key Laboratory of Catalysis and Energy Materials Chemistry of Ministry of Education & Hubei, Key Laboratory of Catalysis and Materials Science, School of Chemistry and Materials Science, South-Central University for Nationalities, Wuhan 430074, China; 2 School of Chemical Engineering and Light Industry, Guangdong University of Technology, Guangzhou 510006, China

**Keywords:** Catalysis, Organic Chemistry, Physical Organic Chemistry, Molecular Electrochemistry

## Abstract

Aryl carboxylic acids are stable and readily available in great structural diversity both from natural and well-established synthetic procedures, which make them promising starting materials in organic synthesis. The conversion of benzoic acids into high-value molecules is of great importance and have gained much interest of synthetic chemists. The recent development of single-electron (1e^−^) activation strategy has been esteemed as a complementary method for the transformation of benzoic acids. In this context, carboxylate groups can be selectively transferred into reactive aryl carboxylic radical, aryl radical, and acyl radical by electrocatalysis, photocatalysis, or in the presence of some SET oxidants. Based on these radical species, remarkable advancements have been achieved for the rapid formation of various chemical bonds over the past 10 years. In this review, we summarize recent advances in single electron activation of aryl carboxylic acids, with an emphasis on reaction scope, catalytic system, limitation, and underlying reaction mechanism.

## Graphical Abstract

**Figure:**
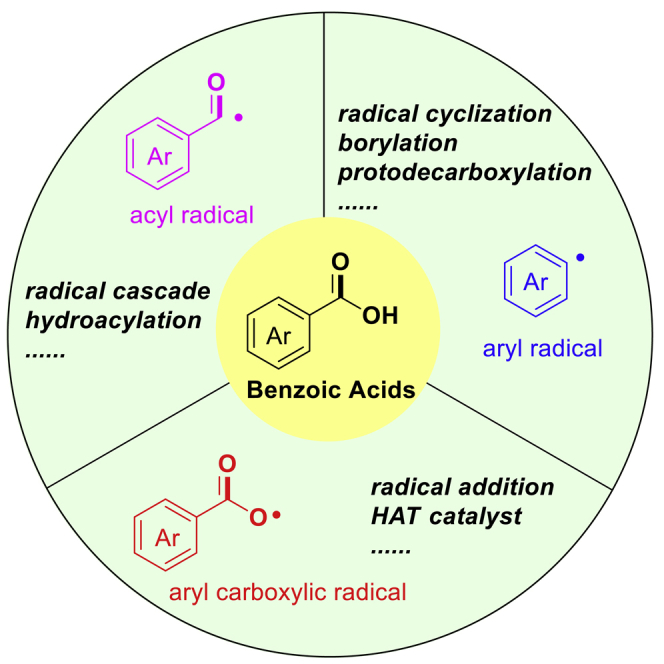

## Introduction

**Scheme 1:**
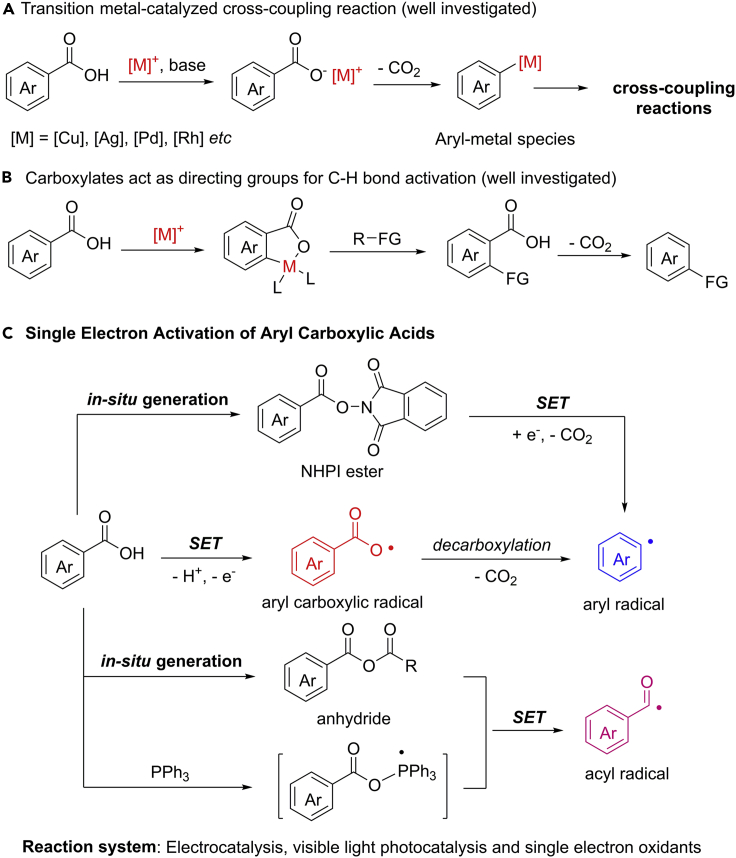
Summary of Classic Reactions and Single Electron Activation of Aryl Carboxylic Acids

Aryl carboxylic acids have been long established as versatile building blocks for the construction of various chemical bonds (Gooβen et al., 2008). Tremendous efforts have been devoted to the important field of decarboxylative functionalizations of benzoic acids toward a wide array of valuable transformations. Transition metal (Ag, Cu, Pd and Rh)-catalyzed decarboxylative cross-coupling reactions provide a powerful platform for the utilization of benzoic acids, in which reactive aryl−metal species are generated *in situ* with the extrusion of CO_2_ from the carboxylate groups (Scheme 1A) (Wei et al., 2017; Font et al., 2017; Patra and Maiti, 2017; Rodríguez and Gooβen, 2011; Weaver et al., 2011). Over the past decades, significant advancements have been made in this field by the groups of Gooβen (Goossen et al., 2006), Larrosa (Cornella and Larrosa, 2012), Glorius (Wang et al., 2010), Miura (Takamatsu et al., 2017), Su (Hu et al., 2012), and You (Tan et al., 2018). However, representative strategies for the decarboxylative process, to a great extent, rely on a relatively high temperature or *ortho*-substituent in benzoic acids, which limited their broad application in practical synthesis. Moreover, carboxylates have also been used as tracelessly cleavable directing groups in a wide range of sp^2^ C−H bond functionalizations (Scheme 1B) (Pichette Drapeau and Gooβen, 2016; Ackermann, 2011; Hu et al., 2018a, 2018b; Gao et al., 2020; Li et al., 2019a, 2019b; Arroniz et al., 2014; Giri and Yu, 2008). Despite these impressive achievements, the development of unconventional and much milder strategies through exploration of catalytic modes is extremely important for the transformation of aryl carboxylic acids but still a challenging goal for scientists.

The continuously increasing demand for sustainable synthesis has inspired organic chemists to explore more efficient methods to produce fine and useful chemicals. The recent development of SET oxidation or reduction strategy provides an efficient means for molecule activation in a mild and predictable manner (Prier et al., 2013; Chen et al., 2016a, 2016b; Yi et al., 2017). Not surprisingly, visible-light photocatalysis (Perry et al., 2018; Skubi et al., 2016; Chen et al., 2016a, 2016b; Romero and Nicewicz, 2016; Karkas et al., 2016) and electrocatalysis (Yan et al., 2017; Zhao et al., 2017; Jiang et al., 2018; Jiao et al., 2020; Zeng et al., 2020; Leech and Lam, 2020) represent two ideal protocols for radical reaction invention because of their green and sustainable properties. In addition, some SET oxidants are also been used for the generation of radical species. Typically, aryl carboxylic acids can be converted into aryl carboxylic radical, aryl radical, and acyl radical species enabled by photocatalysis, electrocatalysis, or in the presence of some external SET oxidants (Scheme 1C). The reactions of carboxylic radicals mainly focus on intramolecular radical additions due to the strongly competitive hydrogen atom transfer (HAT) process to regenerate benzoic acids. They could also act as an efficient HAT catalyst for C(sp^3^)−H bond activation. The carboxylic radicals may further undergo a decarboxylative process to form aryl radicals, which are mainly applied in protodecarboxylation, decarboxylative borylation, and radical addition (Felpin and Sengupta, 2019; Qiu et al., 2016). The *in situ* generated *N*-hydroxyphthalimide (NHPI) esters from aryl carboxylic acids can be also transformed into aryl radicals through a sequential 1e^−^ reduction/decarboxylation process (Recupero and Punta, 2007). It should be noted that NHPI esters are stable and easy to synthesis from benzoic acids through a simple condensation process. Moreover, in the presence of dimethyl dicarbonate (DMDC) under basic conditions, benzoic acids are easy to transfer into the corresponding anhydrides, which can be reduced and result in acyl radicals by releasing CO_2_. The acyl radicals have been widely used in visible light-induced radical cascade reactions for the rapid construction of carbonyl compounds and late-stage modification of biologically active scaffolds (Ryu, 2001; Banerjee et al., 2019; Zhao and Mankad, 2019). The radical reactions of benzoic acids generally feature mild condition, broad scope, and good functional group tolerance by taking advantage of the high efficiency and unique reactivity of radical species.

During the past 10 years, significant advances have been achieved in this field. In this review, we will give a brief overview of radical reaction of aryl carboxylic acids. The radical reactions of hetero(aryl) carboxylic acids are also covered in this review. At the same time, the reaction scope, limitation, and mechanism will be discussed in detail. To calibrate the scope of this interesting topic, the discussion is primarily organized based on the reaction of different radical species. Hopefully, this review will provide a useful guideline for researchers to further exploration of efficient catalytic systems and valuable transformations of aryl carboxylic acids.

### Aryl Carboxylic Radical Reaction

**Scheme 2:**
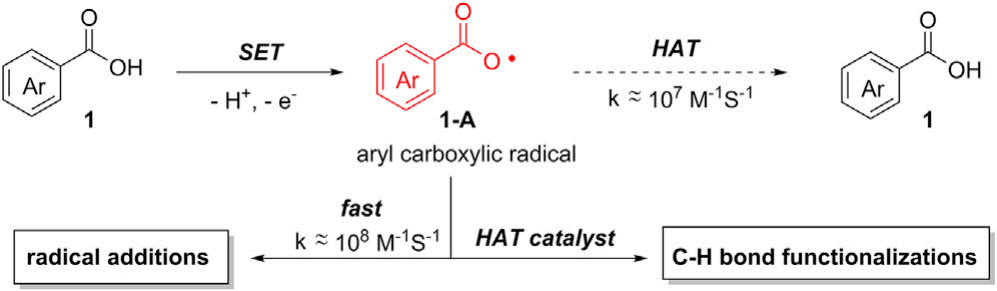
Aryl Carboxylic Radical-Mediated Reaction

Aryl carboxylic acids could be converted into aryl carboxylic radical intermediates via a direct SET oxidation (Scheme 2). The reaction scope of aryl carboxylic radicals is relatively limited and far from matching that of other two types of radicals due to their instability. They may undergo a rapid H−atom abstraction from the reaction system to regenerate benzoic acids. Therefore, the majority of aryl carboxylic radical reactions are intramolecular radical additions. In addition, aryl carboxylic radical is an electrophilic O−centered radical, which can be used as an HAT-catalyst for the functionalization of aliphatic C−H bonds.

**Scheme 3:**
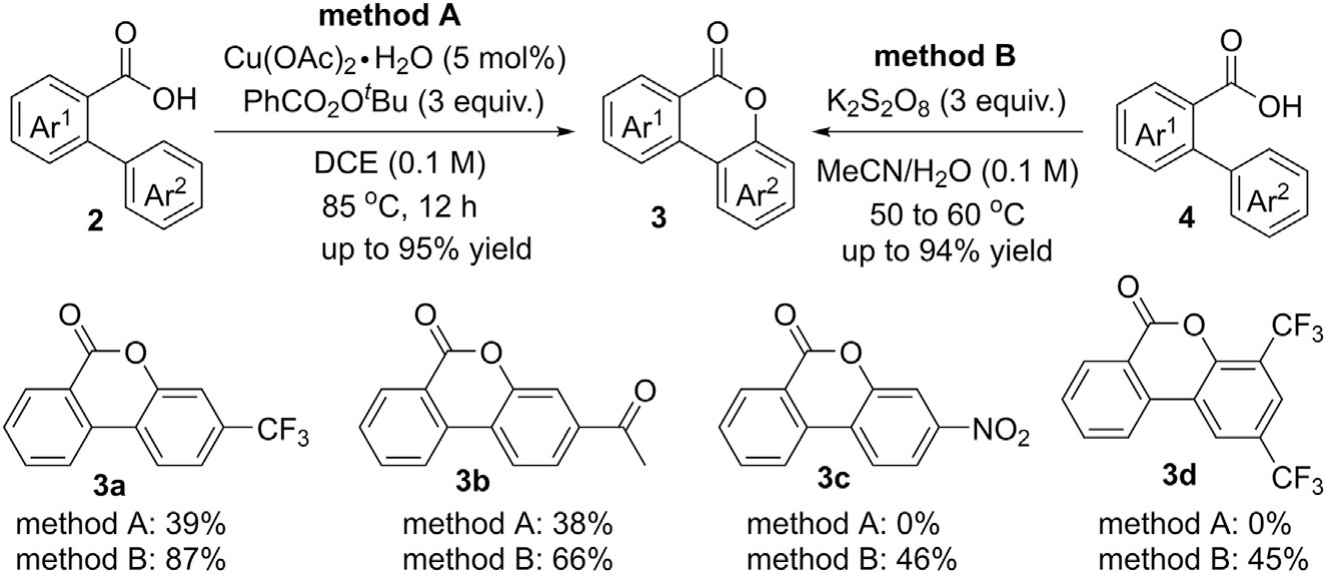
C(sp^2^)−H Oxygenation of Benzoic Acids

In 2013, Martin et al. reported an interesting copper-promoted C(sp^2^)−H hydroxylation assisted by carboxylic acids with the use of benzoyl peroxide (PhCO_2_)_2_ as the oxidant (Gallardo-Donaire and Martin, 2013). In the same year, a similar transformation has been achieved by the group of Gevorgyan (Scheme 3) (Wang et al., 2013). They developed two complimentary approaches for this type of reaction in the presence or absence of copper catalyst. Similar to Martin's work, the electron-poor substrates were less effective than electron-neutral and -rich arenes in current Cu-catalyzed process. To address this limitation, a more general method has been developed by using K_2_S_2_O_8_ as the SET oxidant in a mixture of CH_3_CN and H_2_O, which was suitable for both electron-donating and -withdrawing substituents. It should be noted that the addition of AgNO_3_ has a profound effect on the reaction rates. This reaction provides a straightforward access to densely functionalized benzolactones. Importantly, the final products could be easily converted into biaryl ethers in the presence of MeI and KOH.

**Scheme 4:**
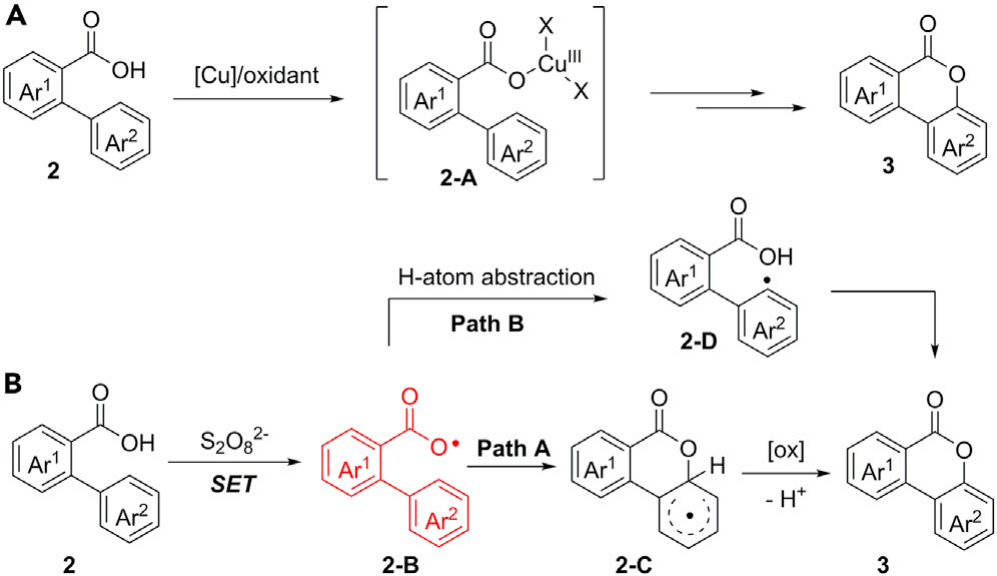
Proposed Mechanism for C(sp^2^)−H Oxygenation of Benzoic Acids

The mechanism of Cu-catalyzed process may proceed through an active Cu(III) species **2-A** (Scheme 4A). In this case, the carboxylate group is coupled with copper catalyst during this reaction. The K_2_S_2_O_8_-mediated pathway most likely involves a free aryl carboxylic radical intermediate **2-B** (Scheme 4B). Firstly, the SET oxidation of benzoic acid provides the key aryl carboxylic radical species **2-B**, which is followed by a rapid radical cyclization to give intermediate **2-C**. Then, **2-C** performs a sequential SET oxidation/deprotonation process to give the final products (Path A). Alternatively, an aryl radical involved mechanism through a hydrogen atom abstraction of radical **2-B** from aromatic ring cannot be completely ruled out at current stage (Path B).

**Scheme 5:**
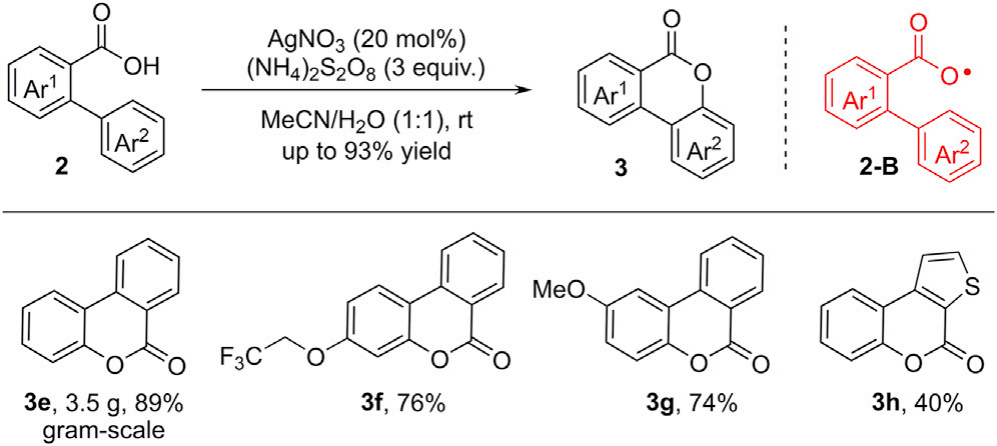
Dehydrogenative Lactonization of Benzoic Acids

Inspired by these works, in 2015, Xu et al. achieved the same reaction by combination of AgNO_3_ as the catalyst and (NH_4_)_2_S_2_O_8_ as a terminal oxidant at room temperature (Scheme 5) (Dai et al., 2015). This protocol can be scaled up to 20 mmol in an open flask, delivering the expected product **3e** in good yield (89%). This reaction features broad scope, simple operation, and good functional group tolerance.

**Scheme 6:**
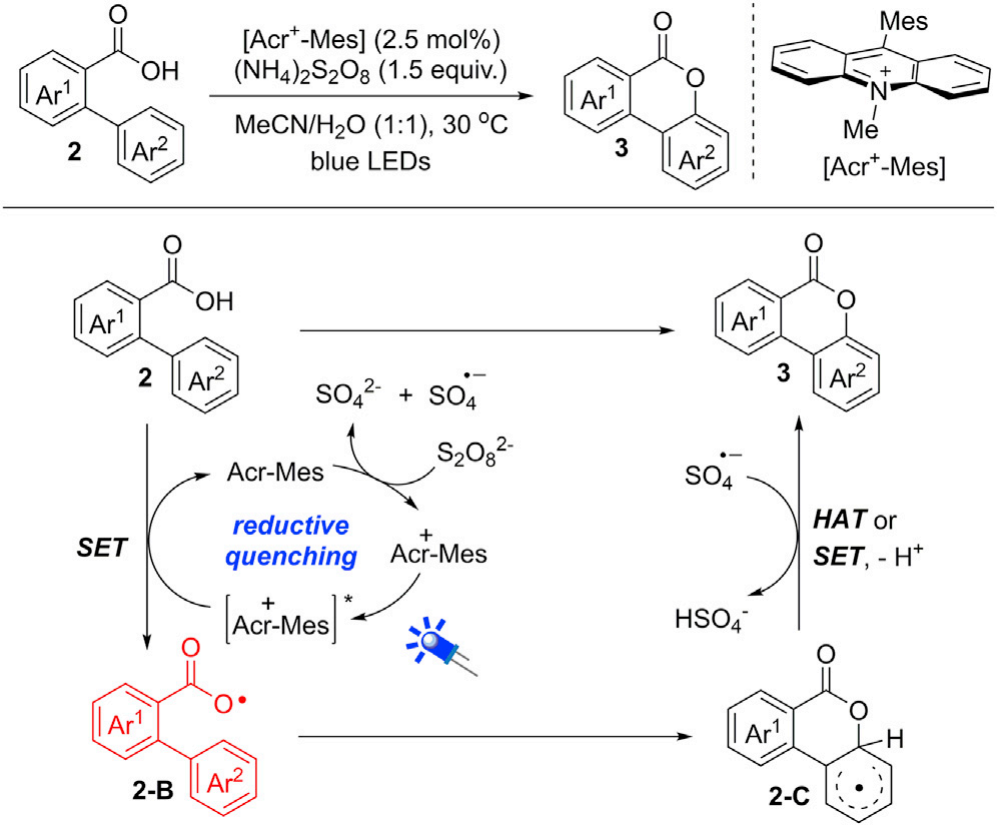
Oxidative Lactonization of 2-Arylbenzoic Acids and Proposed Reaction Mechanism

The recent development of visible light photocatalysis has found widespread application for the generation of active radical species (Yang et al., 2020; Ravelli et al., 2016; Li et al., 2018; Huo et al., 2014; Huang et al., 2020a, 2020b; Yu et al., 2020). In 2015, the group of Gonzalez-Gomez demonstrated an elegant visible light photocatalytic dehydrogenative lactonization of 2-arylbenzoic acids by using [Acr^+^-Mes] (E_1/2_^red^ = +2.06 vs SCE) as a photocatalyst in the presence of (NH_4_)_2_S_2_O_8_ as the oxidant (Scheme 6) (Ramirez et al., 2015). Under the optimized conditions, a wide range of 2-arylbenzoic acids bearing either electron-donating or -withdrawing groups participated well in this reaction. Unfortunately, heterocyclic substrate 2-(3′-pyridinyl)benzoic acid was not suitable. Remarkably, this reaction can be successfully performed upon the irradiation by sunlight.

The reaction was completely inhibited in the absence of visible light or photocatalyst or in the presence of TEMPO. These results suggested a visible-light-promoted radical pathway. Based on the previous work and mechanistic investigations, an aryl carboxylic radical mechanism is proposed in Scheme 6. An SET oxidation of 2-arylbenzoic acid by photoexcited catalyst [Acr^+^-Mes]∗ generates aryl carboxylic radical **2-B**, which then undergoes a 6-*endo*-*trig* cyclization to provide intermediate **2-C.** The sequential SET oxidation/deprotonation, or H−atom abstraction of **2-C,** gives rise to the desired product.

**Scheme 7:**
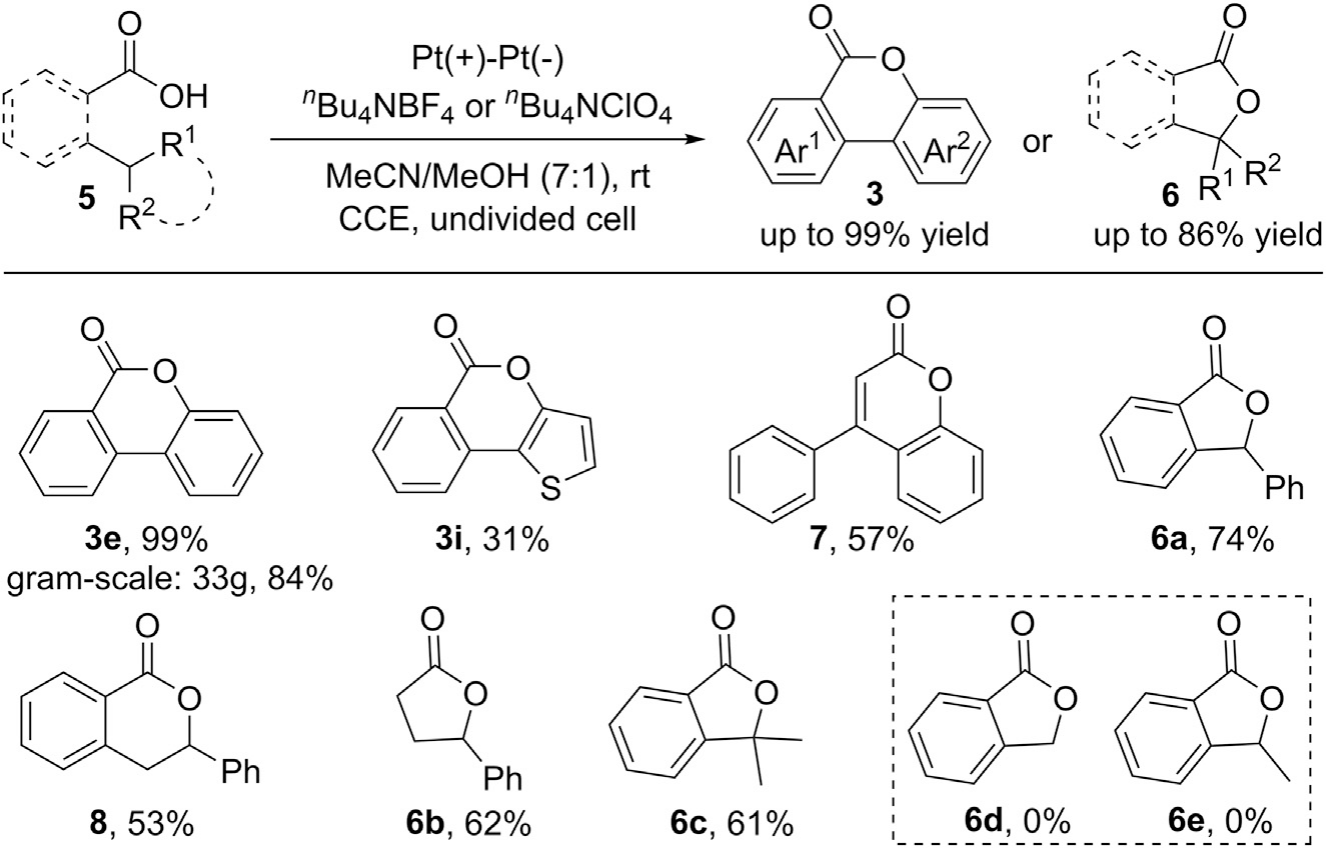
Electrochemical Dehydrogenative Lactonization of 2-Arylbenzoic and 2-Alkylbenzoic Acids

In recent years, the electrocatalysis has witnessed significant advances in radical reactions due to the attractive advantages in terms of low cost, operational simplicity as well as biological tolerance (Kingston et al., 2020; Xiong and Xu, 2019; Ackermann, 2020). In 2017, Zeng and Xu et al. developed an electrochemical dehydrogenative lactonization of aliphatic and aromatic carboxylic acids (Scheme 7) (Zhang et al., 2018a, 2018b). The reaction was conducted in a simple undivided cell with the use of Pt or graphite as the electrodes. A wide range of aromatic carboxylic acids were well tolerated, delivering the diverse lactones in good yield with high regioselectivity. Remarkably, diaryl acrylic acids and 2-alkylbenzoic acids, which proved to be unsuccessful substrates in conventional oxidative conditions, were also compatible in this reaction. For 2-phenethyl benzoic acid, which has two benzylic C−H bonds, the reaction occurred in high selectivity to give isochroman-1-one **8** as a single product. It should be mentioned that a tertiary alkyl substituent is required for the lactonization of simple alkylated benzoic acids due to the instability of *in situ* generated alkyl radical intermediates. The synthetic potential of this transformation was demonstrated by the success of large-scale reaction by using cheap graphite as the electrodes (33 g, 84%).

**Scheme 8:**
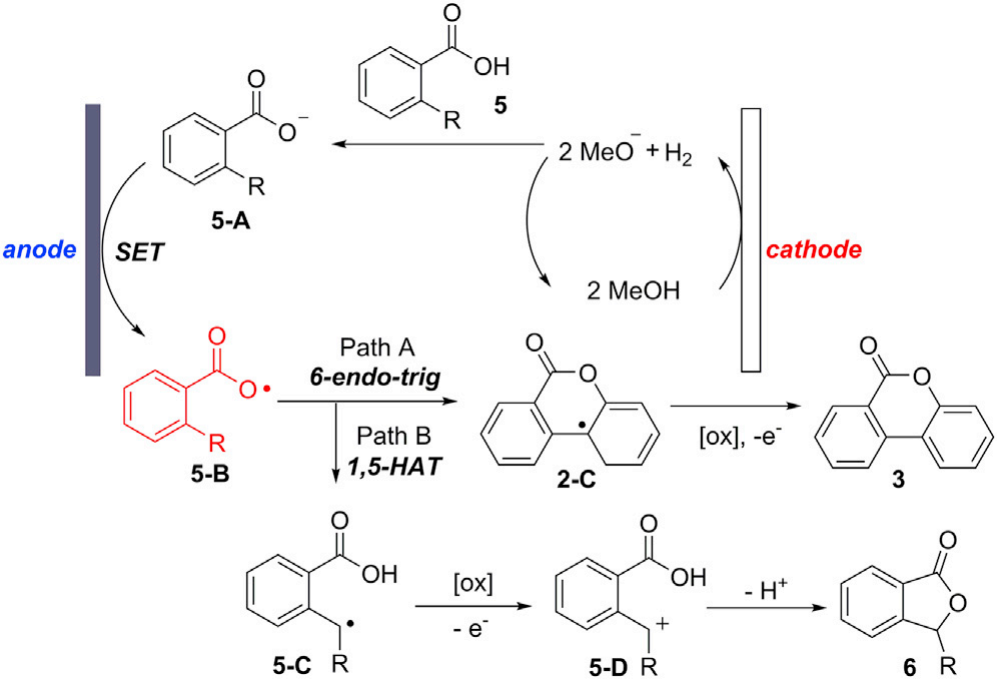
Electrochemical Dehydrogenative Lactonization of 2-Arylbenzoic and 2-Alkylbenzoic Acids

The results of kinetic isotope effect (KIE) studies suggested the cleavage of C(sp^2^)−H bond might not be the rate-determining step. In addition, cyclic voltammetric study demonstrated the oxidation of the carboxylate group (1.4 V versus Ag/AgCl) is favored over aromatic moiety (2.0 V versus Ag/AgCl). On the basis of these observations, a possible mechanism is described in Scheme 8. The mechanism of 2-arylbenzoic acids is similar to the abovementioned visible-light photocatalytic process (Scheme 6). Firstly, the electronic SET oxidation of carboxylate anion **5-A** at anode delivers aryl carboxylic radical intermediate **5-B**, which was identified by the radical trapping experiment by employing 2,2,6,6-tetramethylpiperidine-N-oxyl radical (TEMPO) as the radical scavenger (Scheme 8). Then, the radical **5-B** undergoes a sequential radical addition, SET oxidation, and deprotonation cascade to give the final products. With respect to 2-alkylbenzoic acids, 1,5-HAT process of **5-B** was favored to form a reactive benzyl radical **5-C**, followed by a rapid SET oxidation/intramolecular nucleophilic addition to give the expected products.

**Scheme 9:**
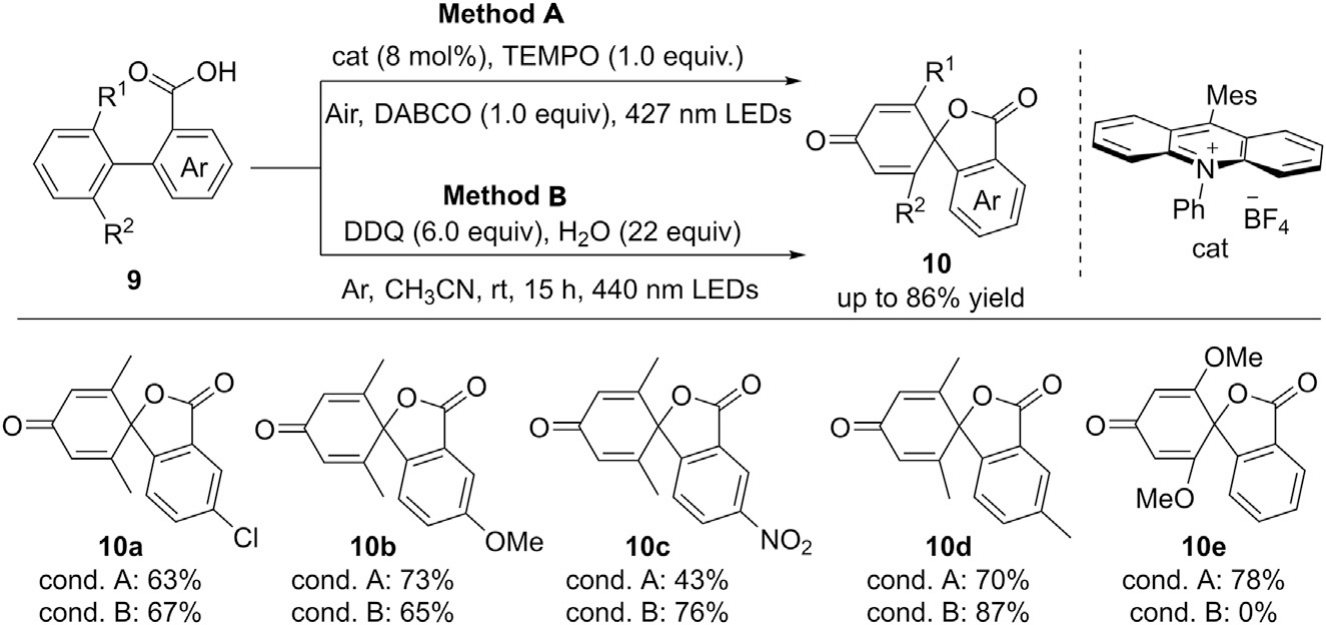
Visible Light-Induced Dearomatization of Biaryl Compounds

Spirolactones exist widely in many natural products and medicinally important molecules (Mao et al., 2017). The development of efficient methods for the assembly of spirolactone motifs has been a field of immense interest. Recently, the group of Samec developed a sustainable process for the construction of functionalized spirolactones via visible-light-induced dearomatization of *ortho*-position blocked biaryl acids (Scheme 9) (Li et al., 2019a, 2019b). It should be noted that the starting material biaryl compounds are readily available from natural lignin. In this reaction, two procedures have been explored. In photocatalytic process, the reaction was conducted at aerobic conditions using an acridinium catalyst as the optimal photosensitizer (Method A). The addition of both TEMPO and 1,4-diazabicyclo[2.2.2] octane (DABCO) significantly improved the reaction efficiency by suppressing the overoxidation pathway. In addition, under anaerobic conditions, 2,3-dichloro-5,6-dicyano-1,4-benzoquinone (DDQ) could act as both a photosensitizer and an SET oxidant to promote this reaction (Method B). These two catalytic systems are complementary to each other. For electron-deficient biaryls with high oxidation potential, better results can be obtained using Method B. In the case of dimethoxy-substituted biaryls, Method A showed higher reaction efficiency.

**Scheme 10:**
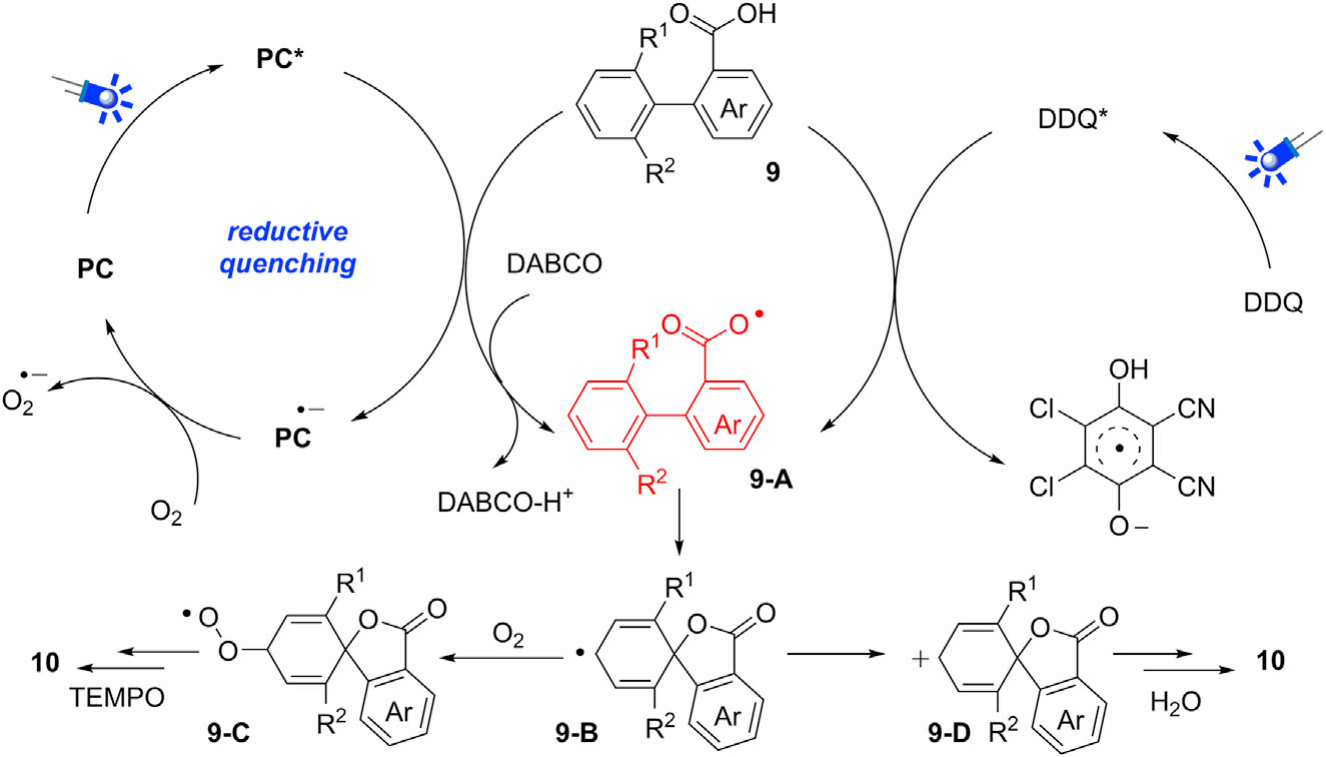
Proposed Mechanism for Radical Dearomatization of Biaryl Compounds

To understand the reaction mechanism, several control experiments have been conducted. Firstly, no product could be obtained in either reaction system under dark condition, which suggested a visible-light-induced pathway. In addition, the different origin of O−atom in dienone moiety was confirmed by labeling experiments, indicating two distinct radical pathways. Moreover, the key aryl carboxyl radical intermediate could be identified by the lactonization of a biphenyl acid under both reaction conditions. Taken together, a possible mechanism is proposed in Scheme 10. In photocatalytic process, the aryl carboxylic radical **9-A** can be generated from an SET oxidation of the carboxyl group by excited photocatalyst PC∗ under basic conditions. The intramolecular cyclization of **9-A** affords radical intermediate **9-B**, which reacts with O_2_ in the presence of TEMPO to give the final product. For DDQ-mediated process, an H−atom abstraction of the carboxyl group by the photoexcited state of DDQ∗ gives the key radical **9-A**. Then, radical **9-A** undergoes an intramolecular cyclization, and SET oxidation cascade gives rise to cation intermediate **9-D**, which is trapped by a molecule of H_2_O and further oxidized to produce the desired product.

**Scheme 11:**
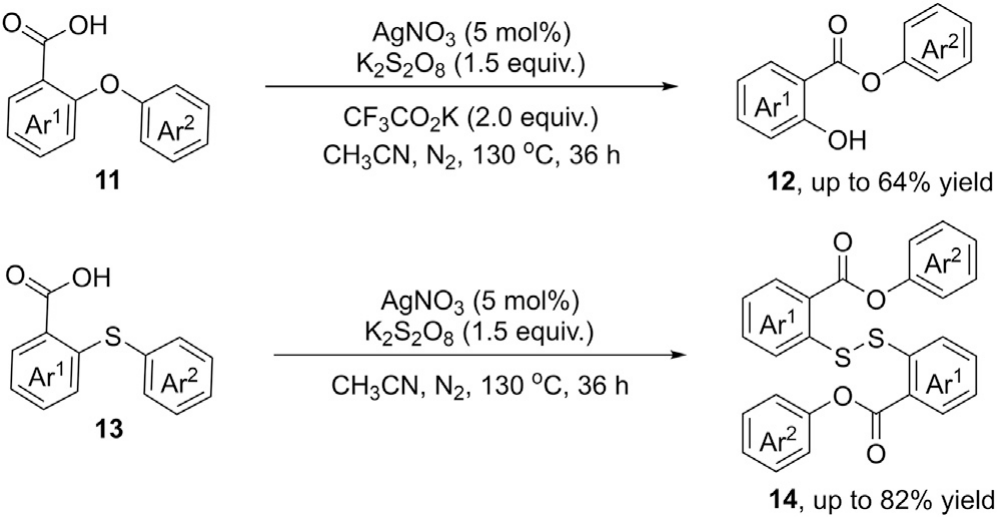
1,5-Aryl Migration of 2-Aryloxybenzoic Acids and 2-(Arylthio)benzoic Acids

In 2016, Jana et al. reported a novel radical 1,5-aryl migration of 2-aryloxybenzoic acids and 2-(arylthio)benzoic acids via silver-catalyzed cleavage of C−O and C−S bonds (Hossian and Jana, 2016) (Scheme 11). In the presence of K_2_S_2_O_8_ (1.5 equiv.) at 130°C, various diaryl ethers reacted smoothly in this reaction, providing the aryl-2-hydroxybenzoate products in satisfying yields. However, the benzoic acid moiety bearing an electron-rich group (5-MeO) failed to give the corresponding product due to the decomposition under this strongly oxidative condition. It is worth mentioning that the reaction efficiency was significantly decreased from 0.1 mmol to 0.5 mmol scale. For the reaction of thioethers, the unusual rearranged disulfide products were obtained through thiyl radical dimerization.

**Scheme 12:**
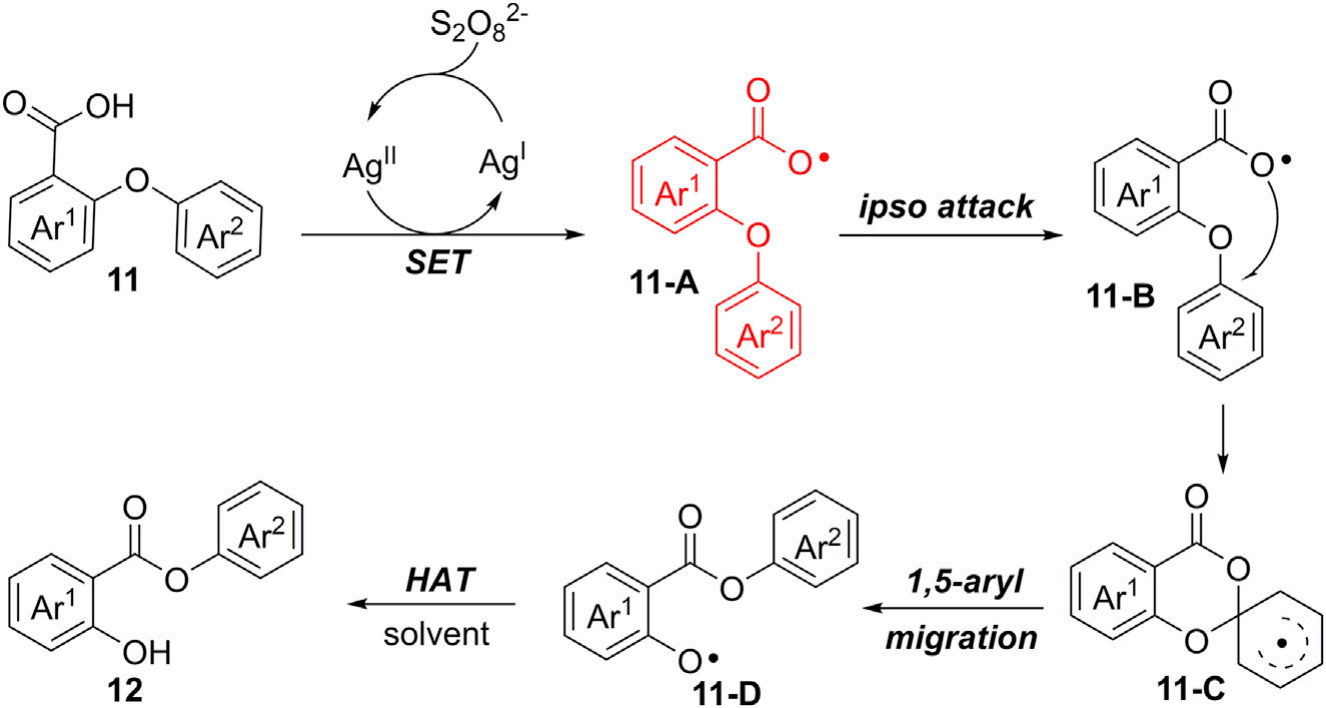
Proposed Mechanism of Silver-Catalyzed 1,5-Aryl Migration Process

The results of radical trapping experiments and cross-over experiments indicated the involvement of a carboxylic radical intermediate and 1,5-aryl migration process in this reaction. Accordingly, a proposed migration mechanism is discussed in Scheme 12. The carboxylate group can be easily oxidized to aryl carboxylic radical **11-A** in the presence of AgNO_3_ catalyst and K_2_S_2_O_8_. Subsequently, an *ipso* attack of **11-A** to arylether moiety forms radical intermediate **11-C**, which proceeds a 1,5-aryl migration process to give phenoxyl radical species **11-D**. Finally, **11-D** abstracts an H−atom from solvent to generate final products.

**Scheme 13:**
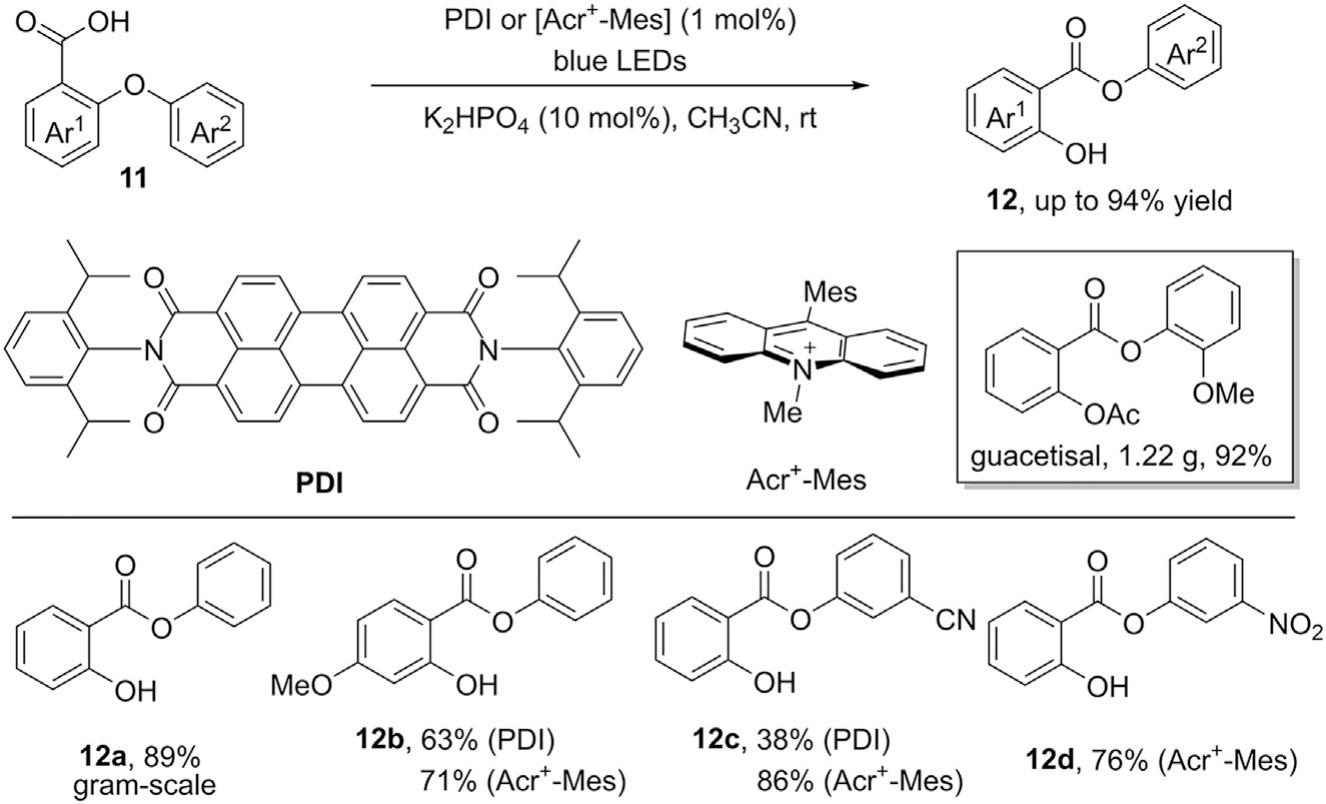
Visible-Light-Induced 1,5-Aryl Migration

In 2017, Li and Cao et al. achieved the similar Smiles rearrangement by visible-light photocatalysis at room temperature (Scheme 13) (Wang et al., 2017). This reaction avoids the use of transition metal catalysts and stoichiometric amounts of oxidant and base. PDI and [Acr^+^-Mes] proved to be two optimal photocatalysts. Upon the irradiation by blue LEDs, a wide range of aryl ethers reacted smoothly to give the desired products in good yields. Significantly, the electron-rich substituents are well tolerated in this reaction, which are unsuccessful substrates under Jana's reaction condition. With the substrate bearing a strongly electron-deficient group (CN and NO_2_), the use of [Acr^+^-Mes] gave the corresponding products in higher yields than PDI. In addition, this protocol can be successfully scaled up to a gram-scale, delivering the expected product in excellent yield. To further demonstrate the synthetic potential of this methodology, a simple one-pot reaction has been developed for the construction of guacetisal in a gram-scale (92%), which has been applied for the treatment of inflammatory respiratory diseases.

**Scheme 14:**
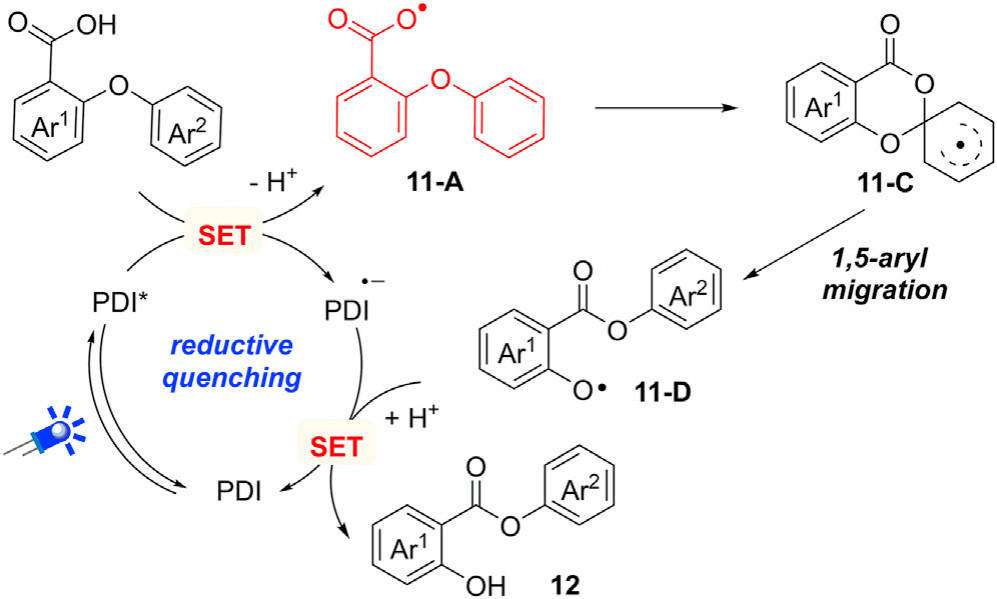
Possible Mechanism for Visible-Light-Induced 1,5-Aryl Migration

The radical trapping experiments by 2,6-di-tert-butyl-4-methylphenol (BHT) and TEMPO indicated a radical mechanism. Meanwhile, the intermediacy of aryl carboxylic radical was demonstrated by EPR studies. Therefore, a carboxylic radical-mediated pathway is outlined in Scheme 14. The reaction starts with the generation of aryl carboxylic radical **11-A** in the presence of K_2_HPO_4_, through an SET oxidation of the carboxylate group (E = +1.90 V versus SCE in CH_3_CN) by the photoexcited catalyst PDI∗ (E = +1.87 V versus SCE in CH_3_CN). Radical **11-A** proceeds a cyclization and 1,5-aryl migration to give O−centered radical **11-D**, which can be reduced by low-valent catalyst species to produce the final product and turn over the photocatalytic cycle. However, on the basis of light on-off experiments and quantum yield value (Φ = 0.24), the author cannot rule out a radical chain pathway of this reaction.

**Scheme 15:**
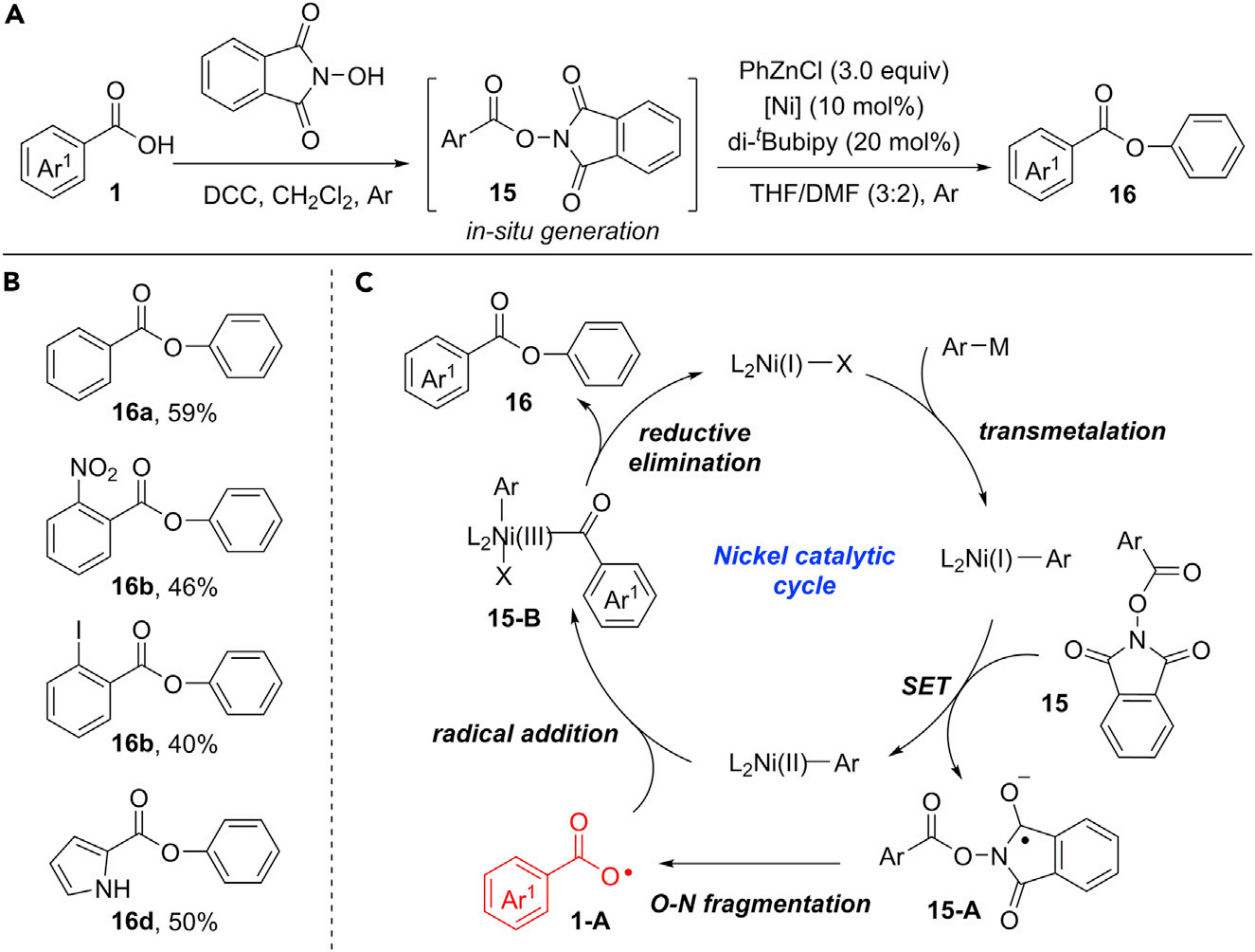
Aryl Carboxylic Radical-Mediated C−O Cross-Coupling Reaction

Recently, Lee et al. developed a novel and convenient aryl carboxylic radical-mediated C−O cross-coupling of NHPI esters with aryl zinc reagents by nickel catalyst under very mild conditions (Scheme 15) (Shih et al., 2019). Importantly, the one-pot reaction from aryl carboxylic acid proceeded very well through the *in situ* generation of NHPI ester. Under current conditions, a wide range of benzoic acids bearing electron-donating and -withdrawing groups at different positions reacted smoothly to produce the desired compounds in moderate to good yields. The scalability and practicability of this protocol was demonstrated by an efficient gram-scale reaction. Initially, the transmetalation of Ni(I)-catalyst with aryl zinc reagent gives rise to a Ni(I)−Ar complex. Then, an SET reaction between Ni(I)−Ar and NHPI ester **15** affords a radical intermediate **15-A** with the generation of Ni(II)−Ar complex. The O−N fragmentation of radical **15-A** gives an aryl carboxylic radical **1-A**, which was confirmed by EPR analysis. The addition of radical **1-A** to Ni(II)−Ar forms a Ni(III)-complex **15-B**. Finally, the reductive elimination of **15-B** gives the product and regenerates Ni(I)-catalyst (Scheme 15C).

**Scheme 16:**
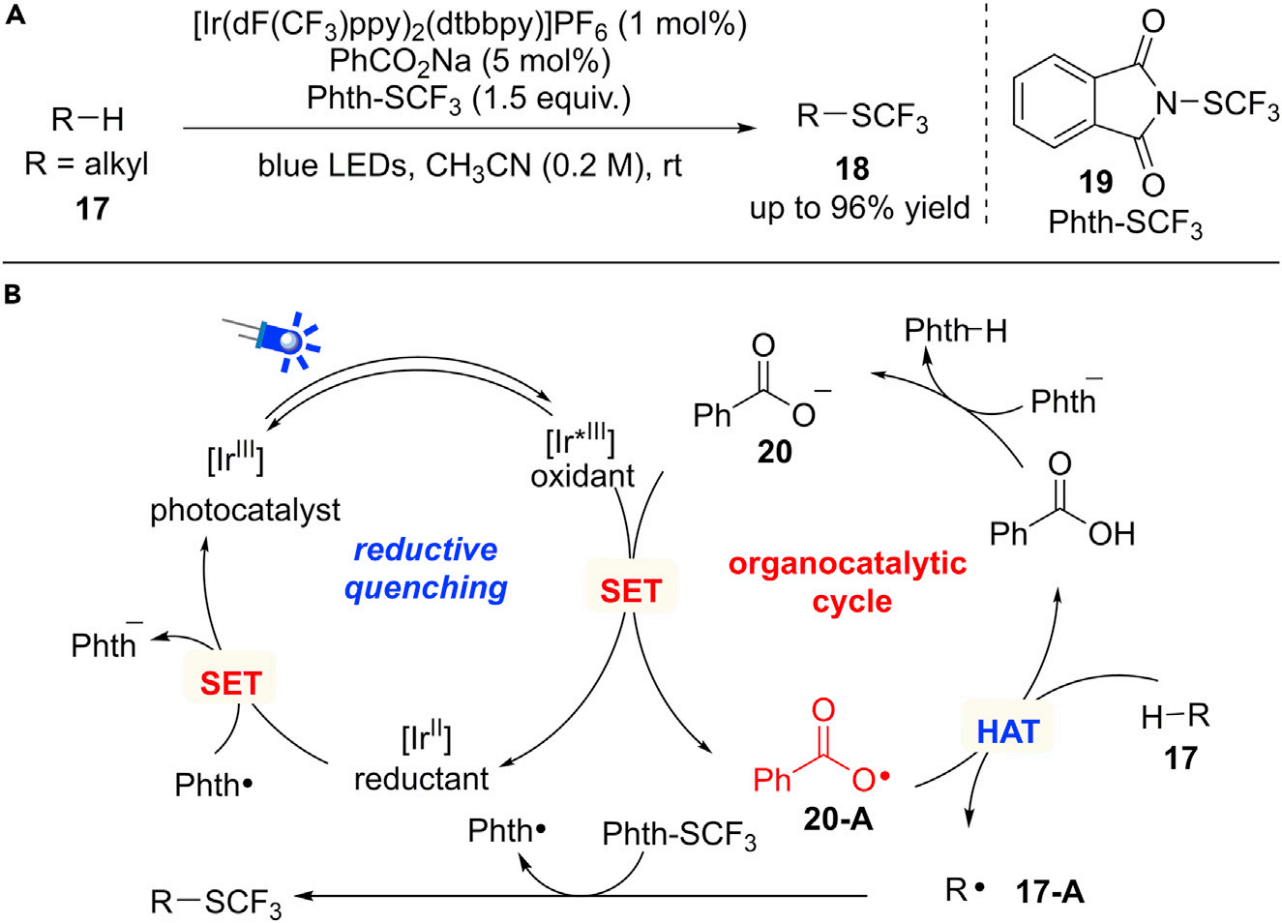
Visible-Light-Induced Trifluoromethylthiolation and Proposed Mechanism

H−Atom transfer is one of the most fundamental reactions, which is involved in many chemical and biological processes. Aryl carboxylic radical is an electrophilic O−centered radical, which can be used as an HAT-catalyst for the activation of aliphatic C−H bonds (Salamone and Bietti, 2015). In 2016, Glorius et al. developed a beautiful *site*-selective C(sp^3^)−H trifluoromethylthiolation by cooperative combination of visible-light photocatalysis and HAT catalysis, using easily available N-(trifluoromethylthio)phthalimide (Phth−SCF_3_) as the SCF_3_-reagent (Scheme 16) (Mukherjee et al., 2016). Sodium benzoate (5 mol%) proved to be the optimal HAT catalyst and [Ir(dF(CF_3_)ppy)_2_(dtbbpy)]PF_6_ was applied as the visible-light photocatalyst. The reaction selectively occurs at tertiary C−H bonds due to the stability of new generated alkyl radical species **17-A**. The protocol shows mild condition, broad scope, good selectivity, and high potential for late-stage modification of biologically active compounds.

Based on mechanistic studies, a carboxylic radical-mediated HAT pathway is proposed in Scheme 16B. The reductive quenching of the highly oxidizing photoexcited state of the Ir(III)∗ catalyst by sodium benzoate **20** results in an aryl carboxylic radical **20-A**. Aryl carboxylic radical is an electrophilic radical, which acts as an HAT catalyst to abstract an H−atom from an aliphatic C(sp^3^)−H bond in alkane substrate **17** by taking advantage of the polarity matching effect to give a nucleophilic alkyl radical **17-A**. Then, radical **17-A** reacts with electrophilic Phth−SCF_3_ to give the desired products with the generation of phthalimide radical (Phth⋅) at the same time. Finally, an SET oxidation of the reduced photocatalyst Ir(II) by Phth⋅ would regenerate the ground state of catalyst and turn over the photocatalytic cycle.

**Scheme 17:**
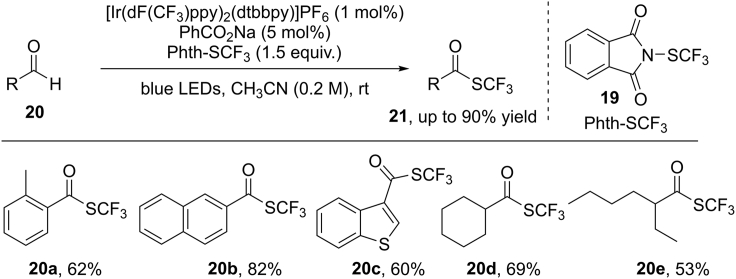
Visible-Light-Induced Trifluoromethylthiolation of Aldehydes

Moreover, Glorius et al. successfully extended the same concept to the selective activation of aldehydic C(O)−H bonds. Accordingly, a visible-light-induced trifluoromethylthiolation of aldehydes has been achieved (Mukherjee et al., 2018) (Scheme 17). In this reaction, both aryl and aliphatic aldehydes were compatible, giving the trifluoromethylthioesters in generally good yields.

### Aryl Radical Reaction

Aryl radicals have been established as a class of well-known and useful radical species and attracted much attention of synthetic chemists because of their wide applications in the construction of various C–C, C–B, C–N, and C–S bonds (Hari and König, 2013; Ghosh et al., 2016; Patra et al., 2013). Typically, aryl radicals are generated from the corresponding aryl halides, aryl hydrazines, arylborates, nitriles, arylsulfonyl chlorides, aryl diazonium, and iodonium salts. In recent years, the oxidative radical decarboxylation of benzoic acids provides an alternative method to generate reactive aryl radical species and have been widely applied in radical arylation reactions.

**Scheme 18:**
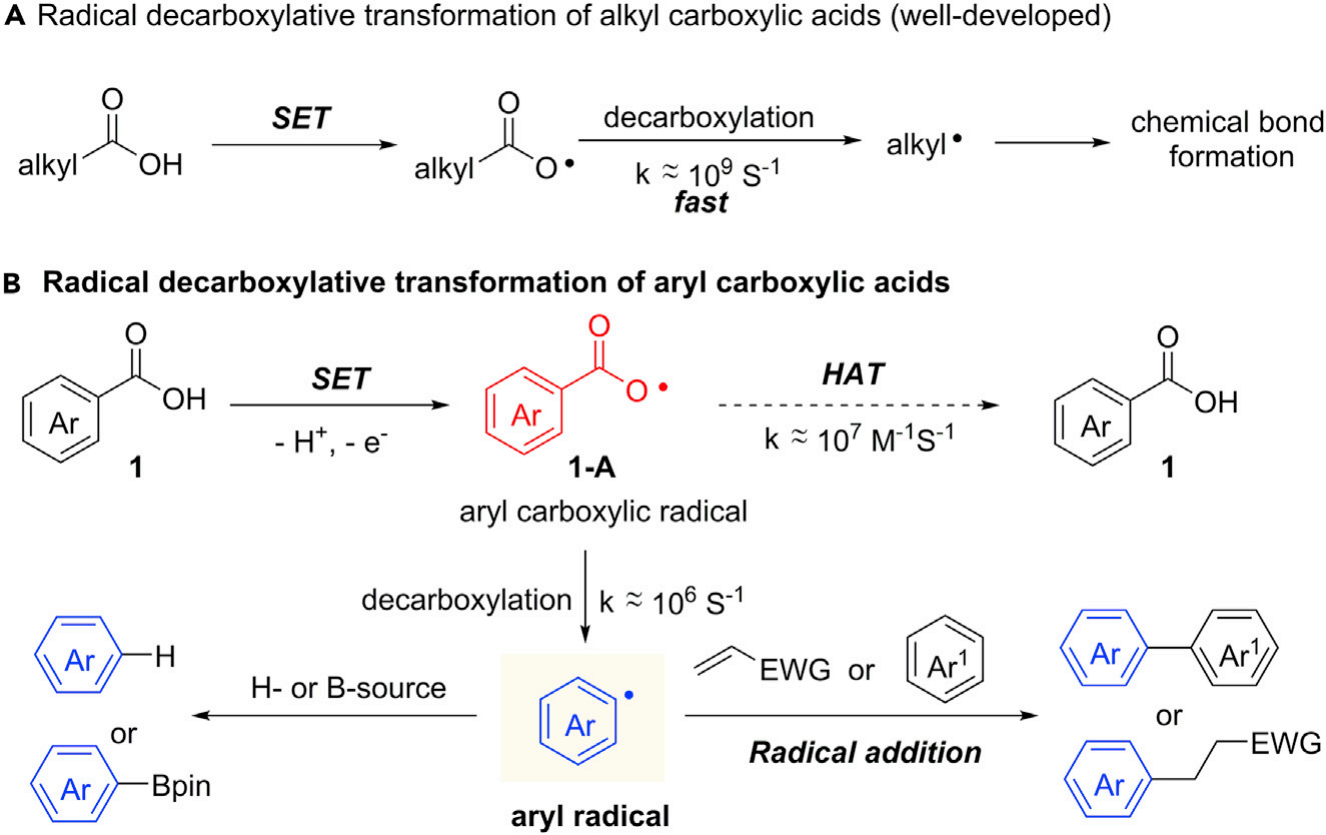
Radical Decarboxylative Transformation of Alkyl and Aryl Carboxylic Acids

Over the past decades, the radical decarboxylative transformations of alkyl carboxylic acids and α-keto acids have been well investigated (Xuan et al., 2015; Trillo and Adolfsson, 2019; Noble et al., 2018; Ventre et al., 2015) (Scheme 18A). The decarboxylation of aryl carboxylic radical is challenging, which has been categorized by Barton as a “non-decarboxylating radical” at temperatures below 130°C (Barton and Ramesh, 1990). The competitive HAT process from solvents or other hydrogen sources is the major challenge in this type of transformations. The rate of decarboxylation (k ≈ 10^6^ s^−1^) is slower than the HAT process (k ≈ 10^7^ M^−1^s^−1^) (Scheme 18B). However, in fact, the decarboxylation of an aryl carboxylic radical is theoretically possible because of the low activation energy (8–9 kcal/mol) (Chateauneuf et al., 1988; Modak et al., 2017). Theoretically, the second step reaction of aryl radical should be fast enough to promote the reaction toward the expected pathway. Therefore, the careful investigation of catalytic system and reaction partners is critical to the success of decarboxylative functionalization of benzoic acids. In this context, the oxidative radical protodecarboxylation, radical addition, and decarboxylative borylation have been developed over the past years.

**Scheme 19:**
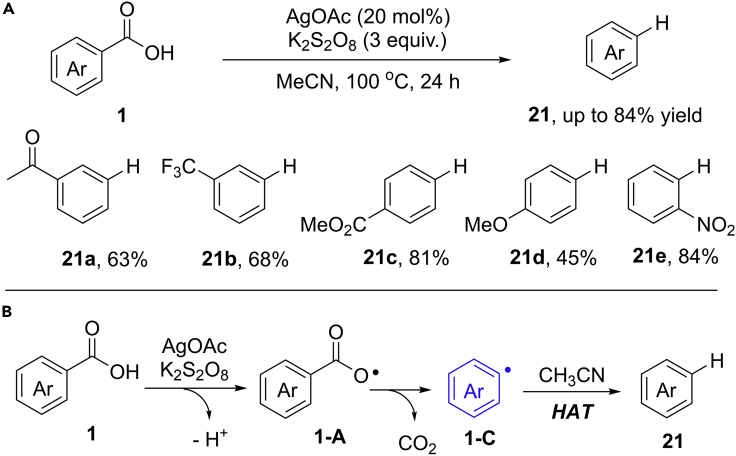
AgOAc-Catalyzed Oxidative Protodecarboxylation of Benzoic Acids

Transition metal-catalyzed (Ag, Cu) protodecarboxylation of heteroaromatic acids have been well investigated by the groups of Goossen and Larrosa (Goossen et al., 2009; Cornella et al., 2009). In this type of reactions, the scope of benzoic acids is generally limited to the *ortho*-substituted carboxylic acids. To address this issue, in 2012, Greaney and co-workers developed an AgOAc-catalyzed oxidative protodecarboxylation of benzoic acids by using K_2_S_2_O_8_ as the oxidant and CH_3_CN as the solvent as well as hydrogen source (Seo et al., 2012a, 2012b) (Scheme 19). In this reaction, a wide range of benzoic acids were well tolerated to give the expected products in generally good yields. However, the benzoic acids bearing an electron-donating group (OMe) gave a lower yield than these electron-deficient benzoic acids due to their slower decarboxylation process.

The transformation is likely to occur via radical pathway rather than a silver-arene intermediate (Scheme 19B). Initially, in the presence of K_2_S_2_O_8_, the Ag(I)-promoted SET oxidation of benzoic acids produces carboxylate radical **1-A**, followed by a decarboxylation process to give the key aryl radical intermediate **1-C**. The subsequent H−atom abstraction of aryl radical from acetonitrile gives the final product.

**Scheme 20:**
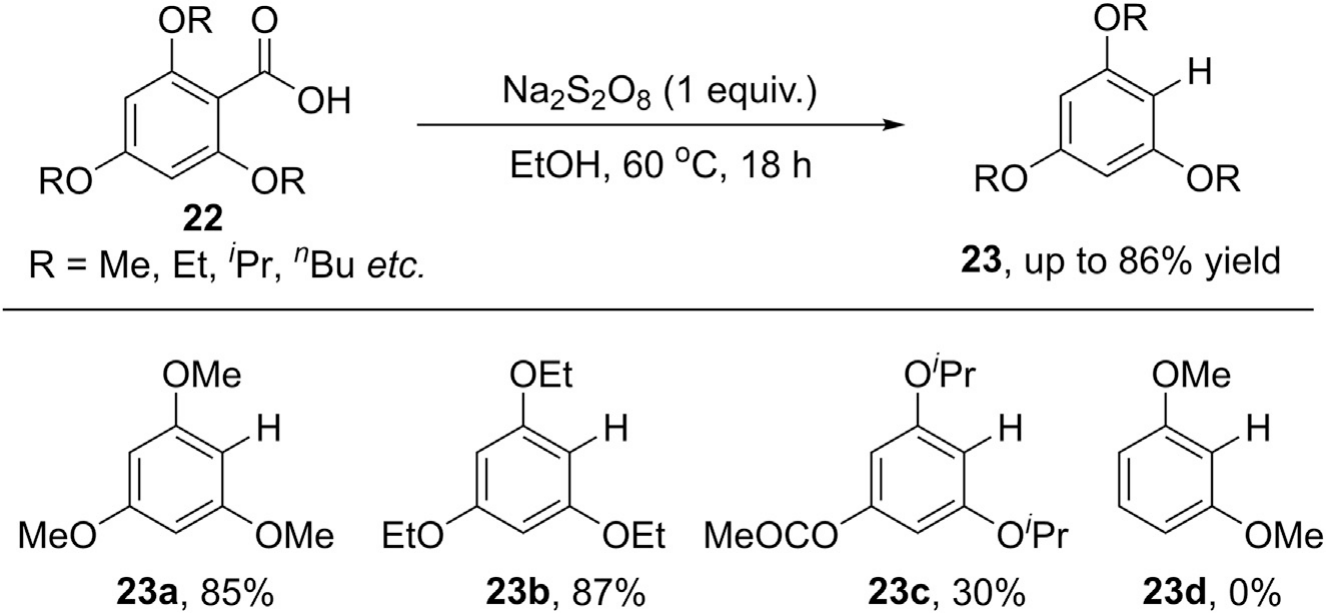
Transition-Metal-Free Protodecarboxylation of Benzoic Acids.

In 2017, Gong et al. further developed a transition-metal free oxidative protodecarboxylation of electron-rich carboxylic acids by employing Na_2_S_2_O_8_ as the external oxidant (Fang et al., 2017) (Scheme 20). This reaction proceeded smoothly at only 60°C in different kind of solvents, such as toluene, ethanol, and dichloromethane. The reaction scope with respect to benzoic acids largely relied on the electronic nature. Trialkoxy-substituted benzoic acids proved to be suitable for this reaction, whereas 2,6-dimethoxybenzoic acid failed to afford the desired product. The change of a methoxy group to acetoxy group sharply decreased the reaction efficiency. More importantly, the author extended this methodology to a decarboxylation/halogenation tandem reaction by using NCS, NBS, or NIS as halogenating reagents. The results of radical inhibition experiments are consistent with a radical decarboxylation pathway of this reaction.

**Scheme 21:**
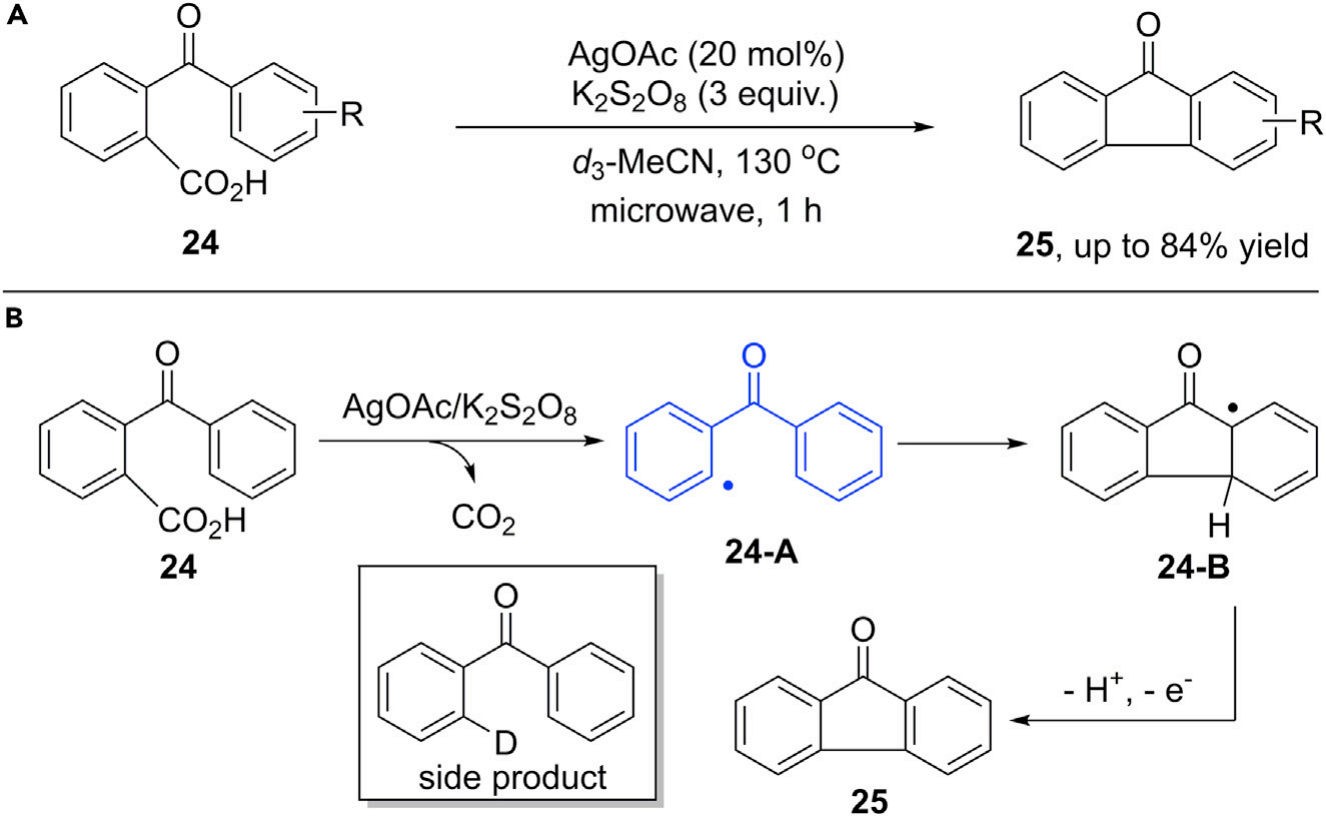
Silver (I)-Catalyzed Decarboxylation/Cyclization Reaction and Reaction Mechanism.

In 2012, the group of Greaney reported an oxidative decarboxylation/cyclization cascade reaction of aroylbenzoic acids for the rapid construction of fluorenones in moderate to good yield (Seo et al., 2012a, 2012b) (Scheme 21). The catalytic AgOAc and stoichiometric K_2_S_2_O_8_ at 130°C have been identified as the best conditions. One of the major challenges in this reaction is the protodecarboxylation pathway, leading to benzophenone byproduct. The unproductive hydrogen atom transfer (HAT) can be suppressed by using deuterated acetonitrile (CD_3_CN) as the solvent instead of acetonitrile. The stronger C–D bond in CD_3_CN can largely suppress the side HAT pathway. The isolation of by-product *d*-benzophenone indicated the radical mechanism of this process. Although there are still some limitations for this protocol, such as high reaction temperature and relatively low efficiency of electron-rich substrates, this work provides an ideal alternative mechanistic approach for classic arylation reactions with extrusion of CO_2_ as the by-product.

The reaction likely begins with the generation of an aryl radical intermediate **24-A** through an oxidative decarboxylation in the presence of Ag(I)-catalyst and K_2_S_2_O_8_. Then, the intramolecular addition of aryl radical to aromatic ring forms a new radical species **24-B**, followed by an SET oxidation and deprotonation process to produce the final product.

**Scheme 22:**
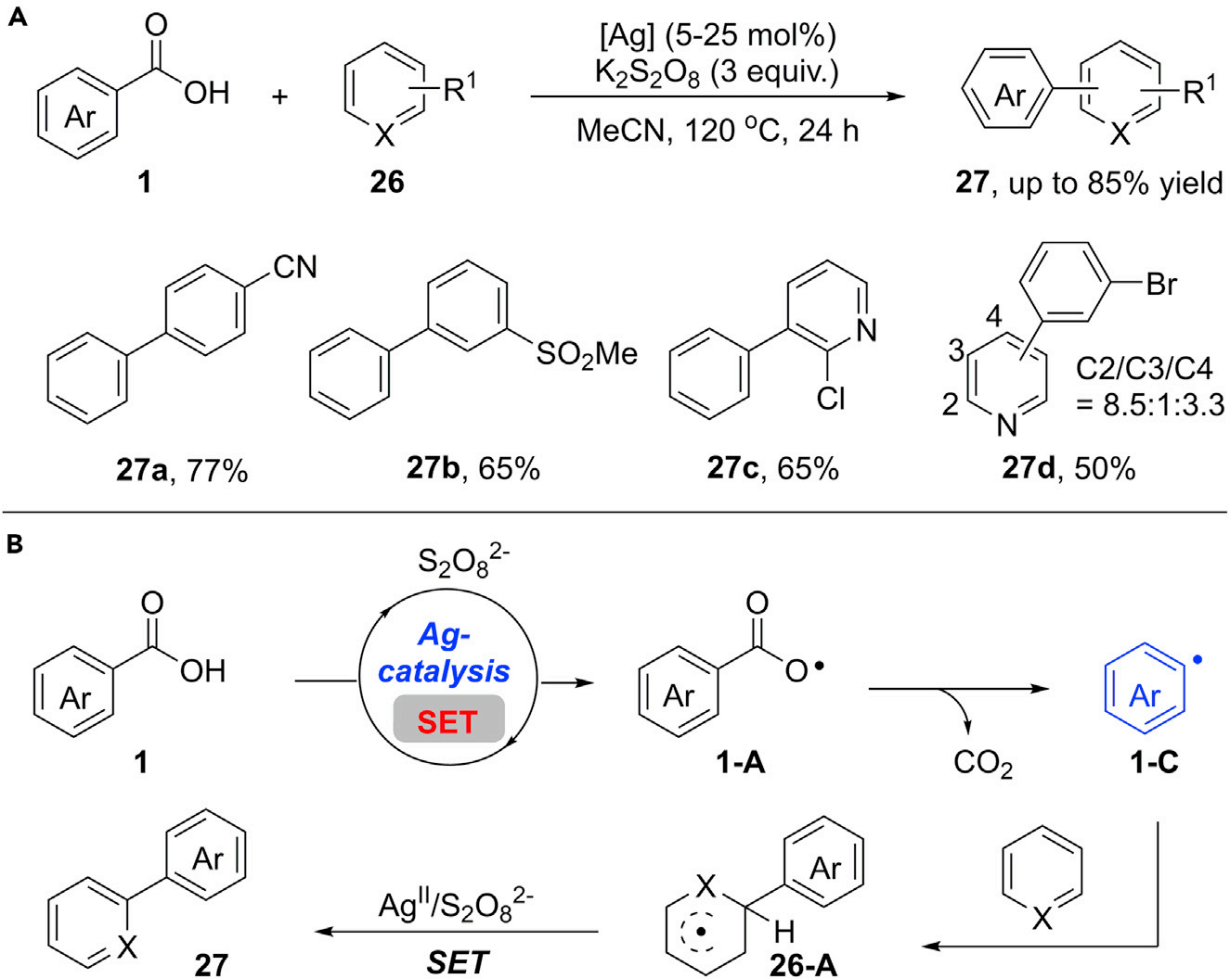
Decarboxylative Arylation of (Hetero)arenes and Proposed Mechanism.

The selective arylation of heteroarenes is a long-standing challenge in the field of Minisci reactions (Proctor and Phipps, 2019). In 2015, Su and co-workers demonstrated an elegant Ag(I)-catalyzed arylation of heteroarenes via radical decarboxylation strategy (Kan et al., 2015) (Scheme 22A). It was found that the loading of silver catalyst was critical for this reaction. When stoichiometric amount of AgOAc was used, trace product can be observed along with a lot of protodecarboxylation by-product. Under the optimal reaction conditions, a wide range of benzoic acids reacted smoothly with various electron-deficient benzenes, furnishing the desired aryl−aryl motifs in generally high yields with moderate to good *site* selectivity. When 22.5 equivalents of TFA was used, pyridine derivatives performed well in this transformation to give the final products in satisfying yields. This reaction provides a new platform for Minisci reaction and a rapid access to aryl−aryl scaffolds.

To further investigate the reaction mechanism, a series of control experiments have been conducted with the addition of radical scavengers such as TEMPO and BHT. As a result, both of these two reactions failed to afford the expected products. In addition, the negligible kinetic isotope effect KIE (k_H_/k_D_ = 1.3) in competition experiment indicated that C–H bond cleavage is not the rate-determining step. These observations agree with a radical pathway. Based on these results, a silver-promoted radical mechanism is proposed in Scheme 22B. Firstly, a single electron oxidation of benzoic acid by Ag(I)/K_2_S_2_O_8_ generates a carboxylate radical **1-A**, which proceeds a decarboxylation process at 120°C to give the reactive aryl radical species **1-C**. Subsequently, the rapid addition of **1-C** to (hetero)arenes produces cyclohexadienyl radical intermediate **26-A**. Then, **26-A** occurs as a sequential SET oxidation and deprotonation procedure to give the product **27**.

Inspired by this work, Qu, Yuan, and co-workers further extended this methodology to the decarboxylative arylation of quinolines under microwave irradiation condition (Yuan et al., 2017). Maiti and Talawar et al. discovered a novel decarboxylative nitration of benzoic acids by using bismuth nitrate as a nitro source and K_2_S_2_O_8_ as the external oxidant at 130°C (Agasti et al., 2019).

**Scheme 23:**
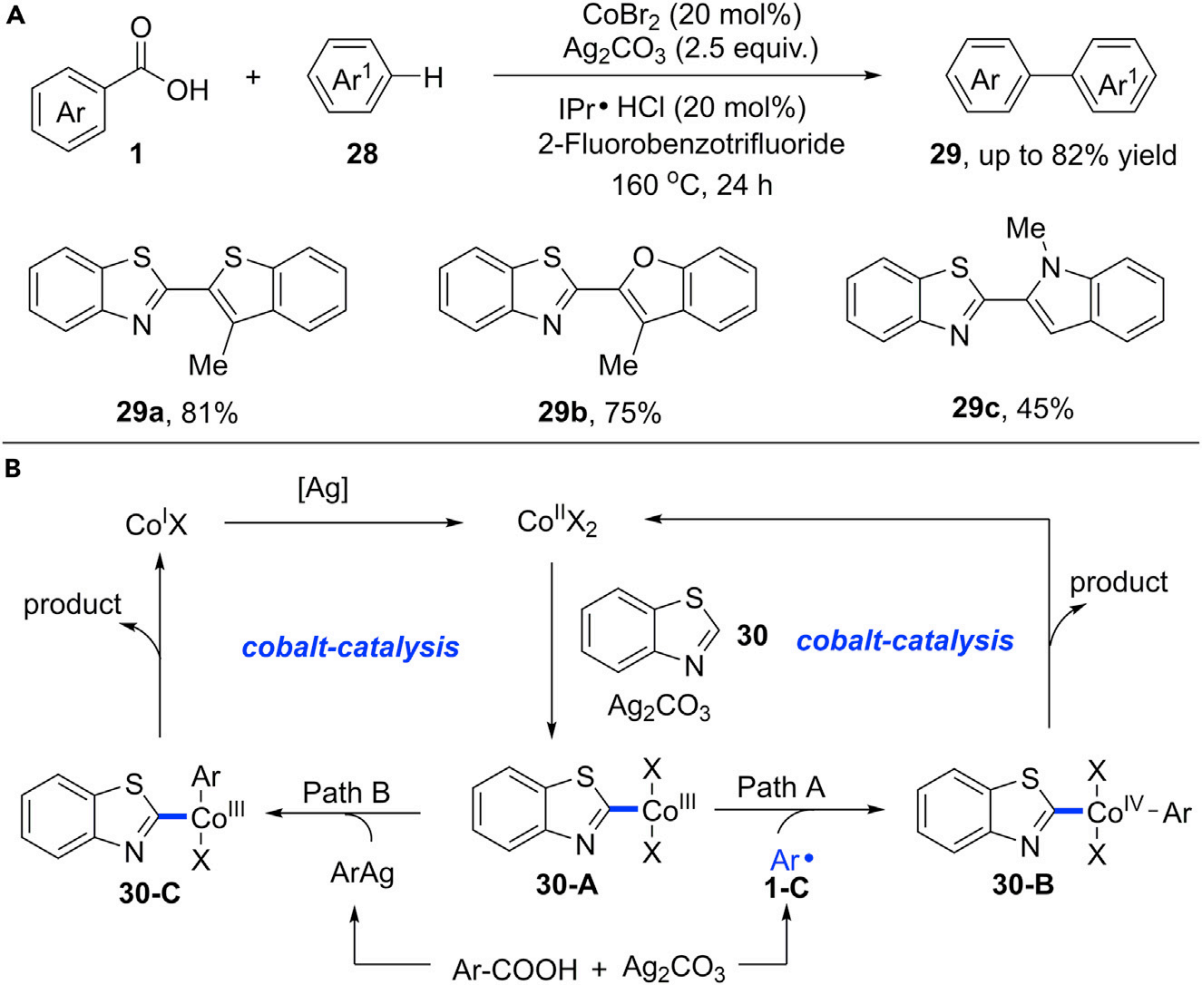
Decarboxylative Arylation of (Hetero)arenes and Proposed Mechanism.

In 2017, Li, Lu, and co-workers reported a cobalt-catalyzed oxidative/decarboxylative C−H arylation of heteroarenes using Ag_2_CO_3_ as the external oxidant in 160°C (Li et al., 2017) (Scheme 23). The addition of N-heterocyclic carbene ligand (Hopkinson et al., 2014) significantly improved the reaction efficiency by minimizing the undesired homo-coupling products. Lower yields were obtained at lower temperature. Various heteroaryl benzoic acids such as 1-methyl-indole-2-carboxylic acid, benzothiophene-2-carboxylic acid, thiazole-5-carboxylic acid, and benzothiazole-2-carboxylic acid participated well in this protocol. The H/D exchange experiments and KIE in parallel experiments suggested that the cleavage of C−H bond in benzoxazoles is reversible and may be a turnover-limiting step. The addition of TEMPO dramatically suppressed this reaction, suggesting a radical pathway.

Before this work, Maiti et al. achieved a copper-catalyzed decarboxylative C–H arylation reaction (Patra et al., 2016). Based on the results of mechanistic study and literature analysis, the author proposed a Co(III/IV/II) catalytic mechanism in Scheme 23B (Path A). Firstly, the oxidation of Co(II) catalyst by Ag_2_CO_3_ gives the reactive Co(III) species. The metallization of benzoxazole via a deprotonation process gives rise to Co(III)-complex **30-A**. At the same time, in the presence of Ag(II) catalyst at 160°C, carboxylic acid undergoes a radical decarboxylation to give aryl radical intermediate **1-C**. The rapid addition of aryl radical to Co(III)-complex generates a new Co(IV)-complex **30-B**. Then, the reductive elimination of **30-B** affords the desired product and regenerates Co(II) catalyst. However, at current stage, a Co(II/III/I) catalytic cycle via silver-mediated decarboxylation, transmetalation, and reductive elimination process cannot be completely ruled out (Path B).

**Scheme 24:**
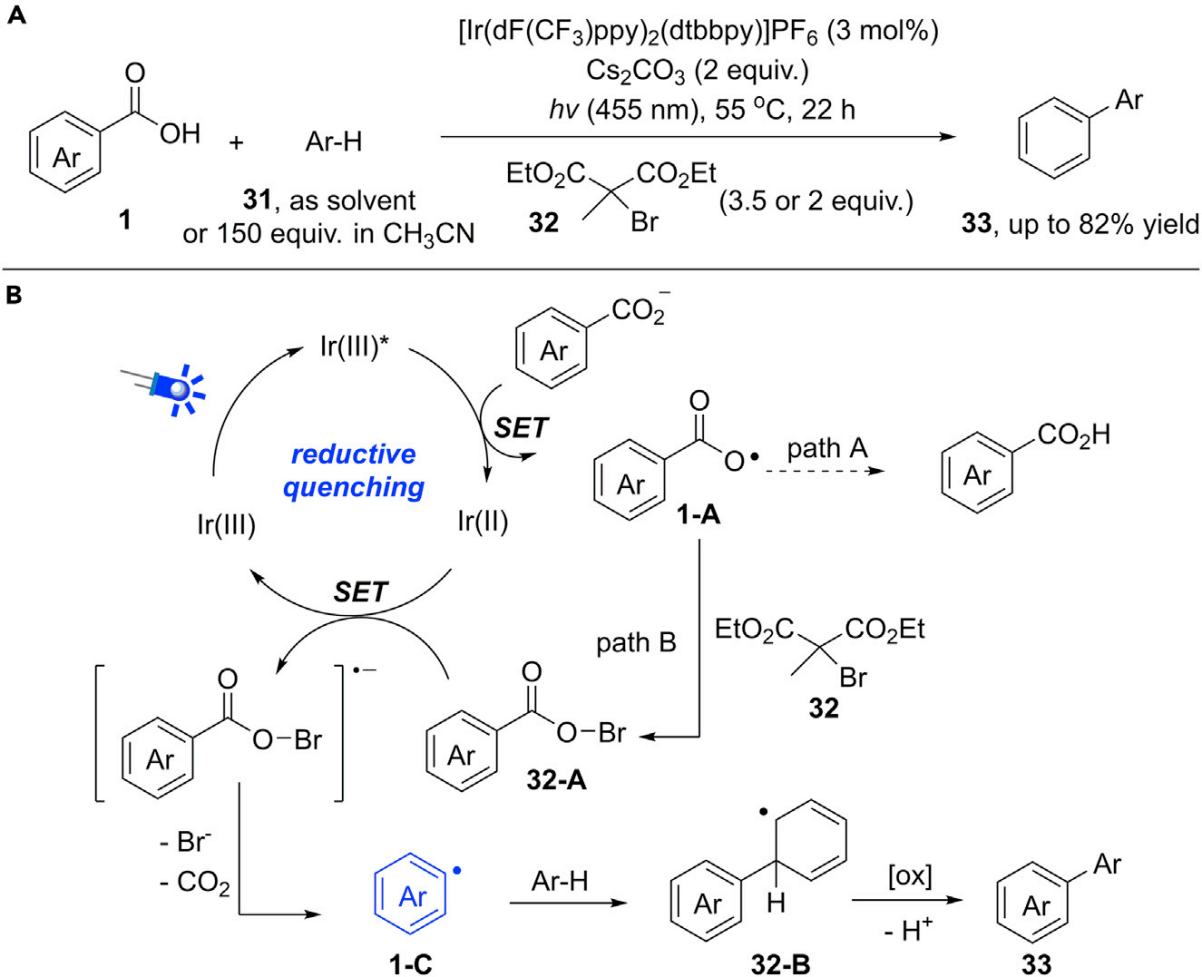
Visible-Light-Induced Decarboxylative Arylation of Arenes and Possible Mechanism.

The visible-light photocatalytic functionalization of benzoic acids is less unexploited and remains a challenging task for organic scientists. As an important breakthrough in this field, in 2017, Glorius et al. disclosed a radical decarboxylation of aryl carboxylic acids for the first time by visible-light photoredox catalysis (Candish et al., 2017a, 2017b) (Scheme 24). The reaction can be achieved at only 55°C with the use of arene as the solvent or 150 equivalents of arene in CH_3_CN (1:1 vol/vol). Notably, the *ortho*-substituents or electron-deficient benzoic acids are not required in this protocol. A brominating reagent is critical for this reaction, which can stabilize the active carboxylic radical by *in-situ* generation of benzoyl hypobromite intermediate. Therefore, the undesired protodecarboxylation and radical addition pathways could be largely suppressed. This reaction provides a new and efficient method for the decarboxylative functionalization of benzoic acids under mild photocatalytic conditions. However, one limitation of this reaction is the requirement of large excess of arenes.

The results of luminesce quenching experiments supported the oxidation of benzoate anion to aryl carboxylic radical by excited photocatalyst Ir(III)∗. In addition, the stoichiometric experiments of hypobromite indicate its intermediacy in this reaction. The proposed mechanism is outlined in Scheme 24B. The reaction begins with the generation of excited state photocatalyst ∗Ir(III) upon irradiation by blue LEDs (455 nm). Then, a reductive quenching of ∗Ir(III) by benzoate anion gives the carboxylic radical **1-A**, followed by a Br−atom abstraction from brominating reagent giving rise to the hypobromite intermediate **32-A**. Then, **32-A** undergoes a sequential SET reduction/decarboxylation process to give aryl radical intermediate **1-C**. Finally, radical **1-C** continues a radical addition, SET oxidation, and deprotonation cascade to deliver the expected product.

**Scheme 25:**
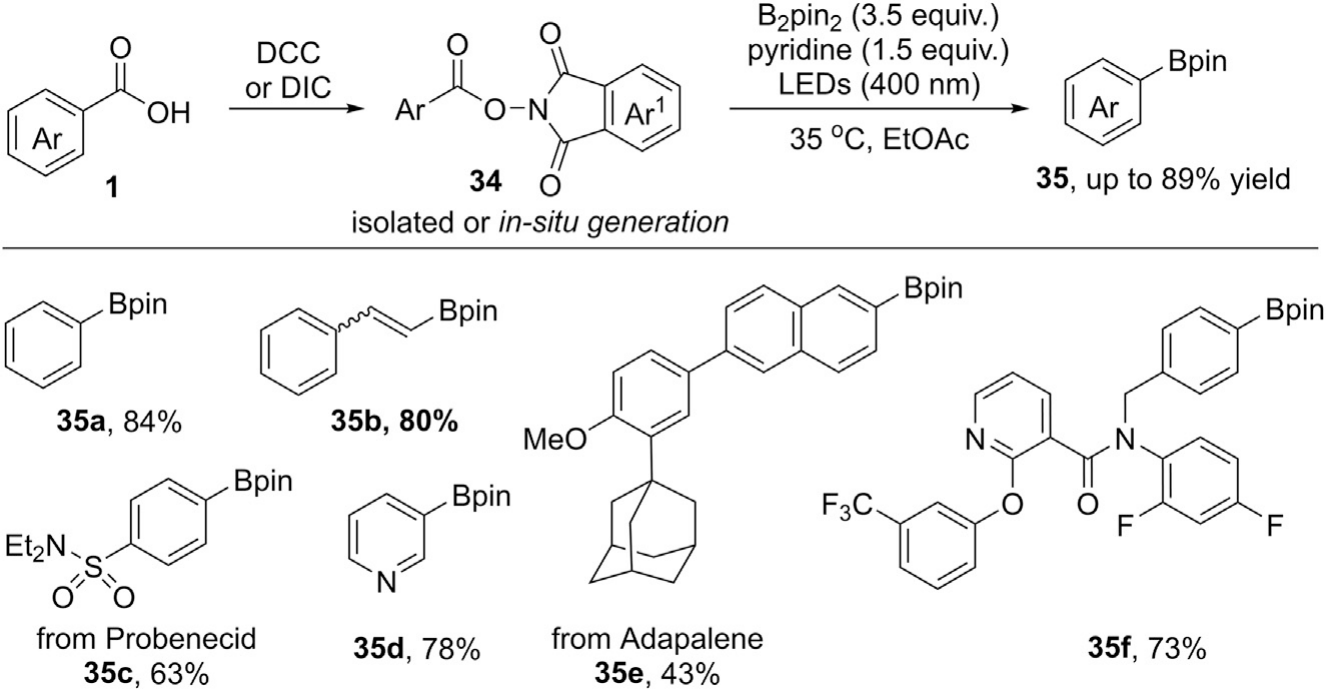
Photocatalytic Decarboxylative Borylation of N-Hydroxyphthalimide Esters.

In the same year, Glorius et al. reported an interesting decarboxylative borylation of NHPI esters under metal-free visible-light photocatalytic conditions (Candish et al., 2017a, 2017b) (Scheme 25). *N*-Hydroxyphthalimide (NHPI) esters are stable and easy to make from commercially available benzoic acids. Alternatively, the NHPI esters could be generated *in situ* in one-pot reaction. The reaction displays simple operation, broad substrate scope, and mild condition. It should be noted that the *ortho*-substituted benzoic acids lead to relatively lower yields due to the low conversion of starting materials and protodecarboxylation by-products. Importantly, this protocol can be applied to the borylation of some biologically compounds such as probenecid and adapalene, furnishing the expected products in 63% and 43% yields, respectively. In addition, the cinnamic acid was also suitable for this transformation.

**Scheme 26:**
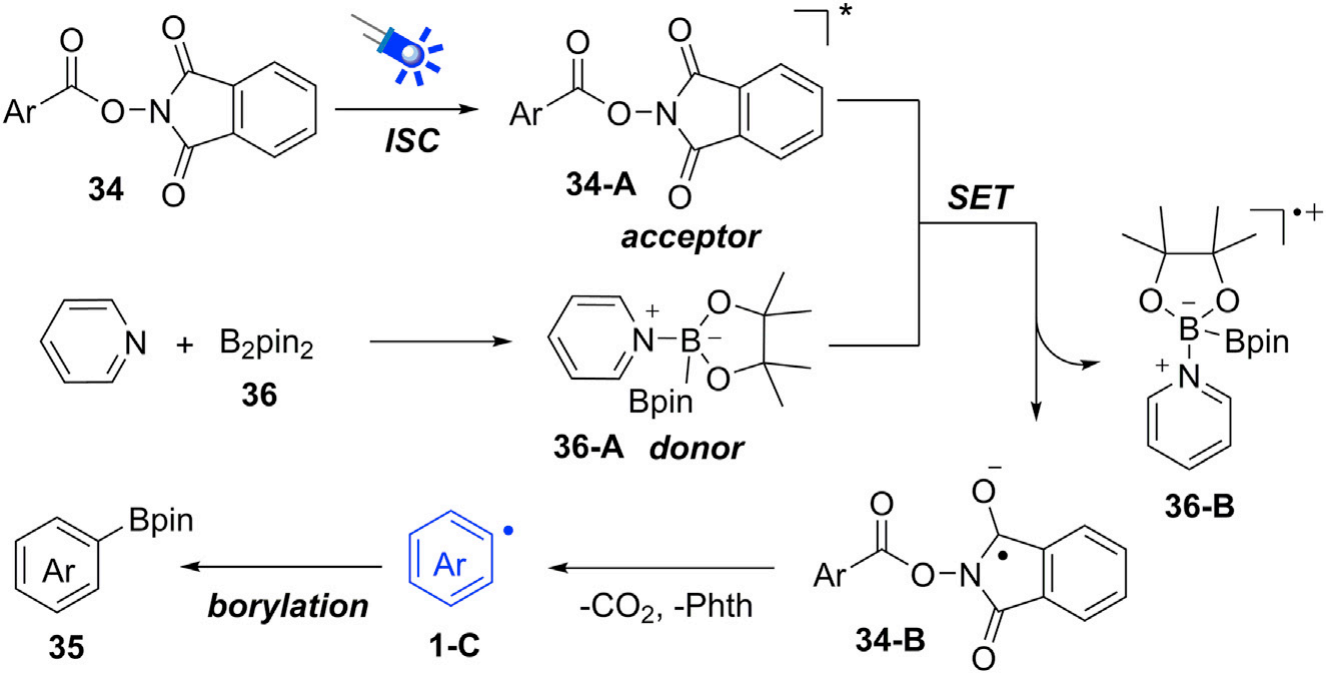
Possible Mechanism of Photocatalytic Decarboxylative Borylation.

The NHPI ester displayed absorption at 400 nm by UV-visible spectroscopic analysis. The measurement of reaction mixture excluded the formation of electron donor-accept (EDA) complex. Moreover, the result of fluorescence quenching experiments supported the SET process between NHPI ester and pyridine-boryl complex. Based on mechanistic studies, a light-induced radical mechanism is proposed (Scheme 26). Under blue LEDs irradiation, the NHPI ester arrives to its excited species **34-A**, which acted as an SET oxidant (E^1/2^ = +1.6 V versus SCE). At the same time, pyridine reacts with diboronate ester to give a donor complex **36-A**. An SET reaction between **34-A** and intermediated **36-A** gives radical anion **34-B** with concomitant of radical cation **36-B**. Then, the radical decarboxylation of **31-B** affords the key aryl radical intermediate **1-C**, followed by a borylation process to give the desired products.

**Scheme 27:**
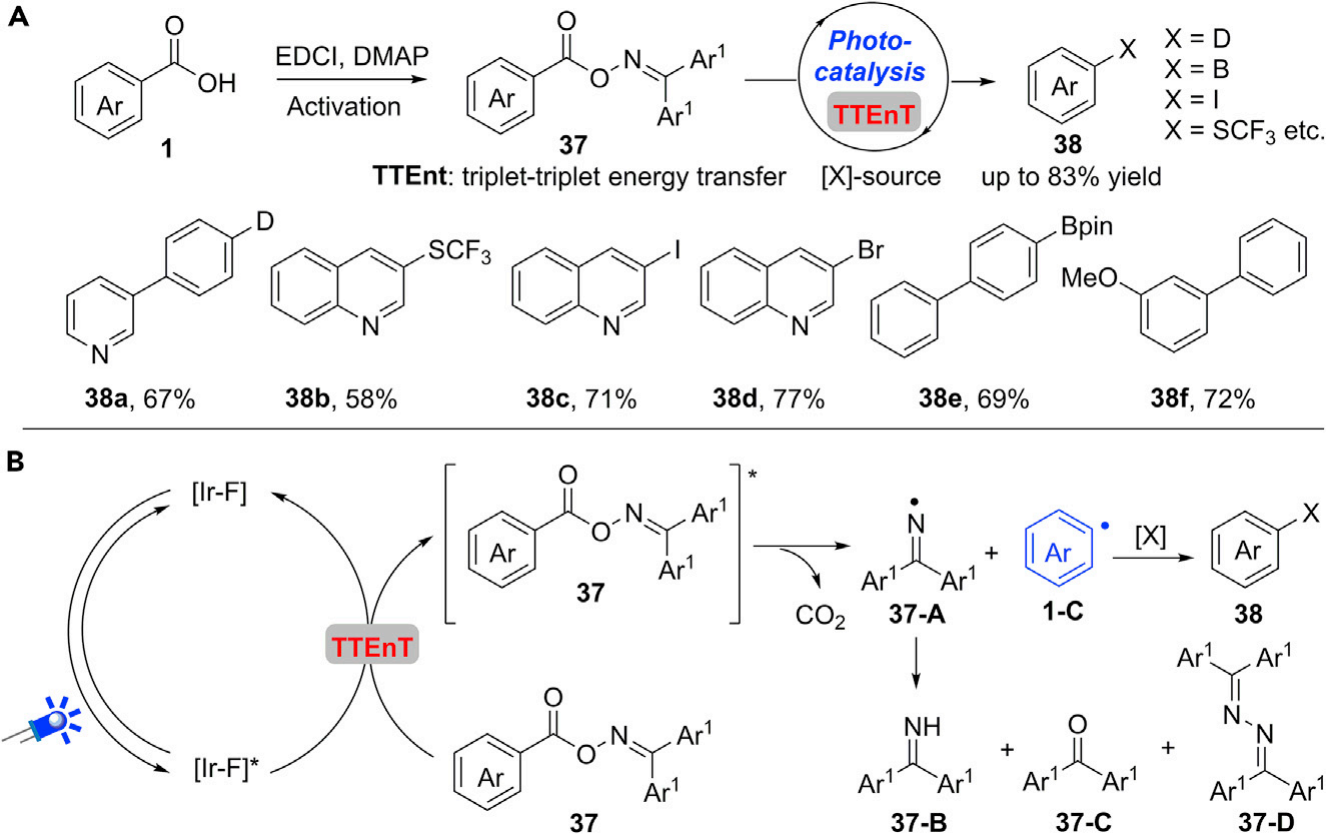
Photocatalytic Decarboxylative Functionalization Reactions.

Very recently, Glorius and co-workers further extended this concept for the decarboxylative deuteration, arylation, bromination, iodination, borylation, and trifluoromethylthiolation by photoredox catalyst (Patra et al., 2019) (Scheme 27A). The choice of a suitable carboxylate group activator is important for this reaction, and aromatic aldoximes derived from carboxylic acids proved to be the best activator. This reaction provides a general and mild method for the rapid formation of various C−C, C−B, C−O, C−I, C−Br bonds. In addition, the scope of this reaction can be extended to aliphatic carboxylic acids. Comprehensive mechanistic studies indicated a triplet-triplet energy transfer (TTEnT)-promoted concerted homolytic N−O bond cleavage and decarboxylation pathway (Scheme 27B). The reaction starts with the generation of triplet excited state of the photocatalyst [Ir-F]∗ upon the irradiation by blue LEDs (λ_max_ = 400 nm), which performs a TTEnT process with substrate **37** to form the triplet excited state **37∗** and regenerate the photocatalyst. Then, **37∗** undergoes a concerted homolytic cleavage to give the aryl radical **1-C** and iminyl radical **37-A**, respectively. The aryl radical **1-C** reacts with suitable trapping reagents to deliver the final products. The iminyl radical **37-A** may participate in an H−atom abstraction, hydrolysis, or dimerization process to give the corresponding by-products **37-B**, **37-C**, or **37-D**.

**Scheme 28:**
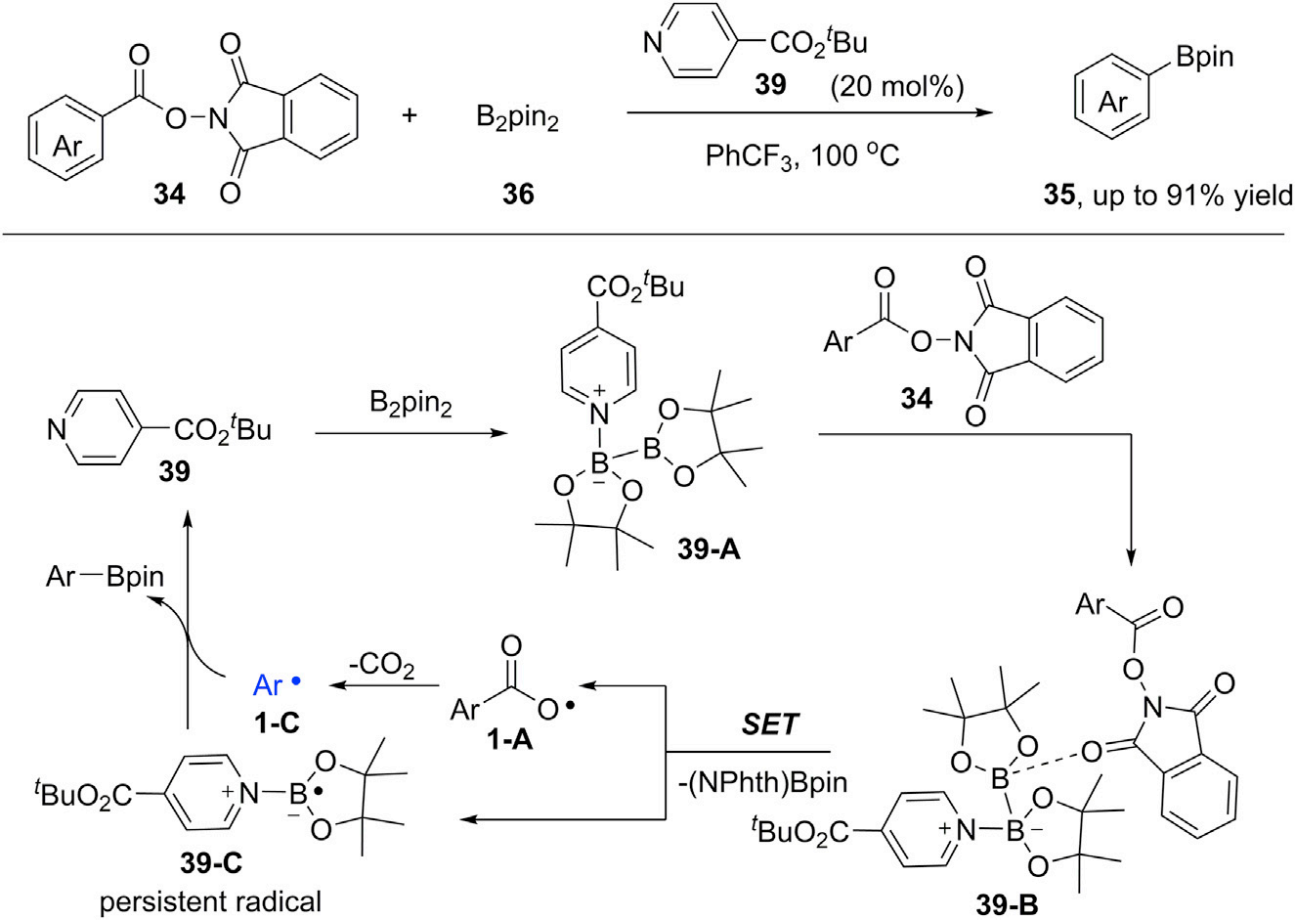
Decarboxylative Borylation of N-Hydroxyphthalimide Esters.

This concept was also used by Fu and co-workers, who realized an interesting decarboxylative borylation under light- and base-free condition (Cheng et al., 2017) (Scheme 28). The electron-deficient isonicotinate *tert*-butyl ester **39** was the best catalyst for activation of diboron reagent. In refluxing PhCF_3_ (100°C), a wide range of NHPI esters was well tolerated, furnishing the desired aryl boronate esters in generally good yields. The current reaction could be easily scaled up to 8 mmol to give the expected product in 85% yield (2.5 g). Notably, diverse transformations of aryl boronate esters have been conducted for the construction of C−CF_3_, C−S, C−N, and C−O bonds. In this reaction, a complex **39-B** of isonicotinate *tert*-butyl ester, diboron reagent, and NHPI ester has been proposed based on ^11^B NMR analysis. Then, the complex **39-B** undergoes a sequential single-electron transfer, decarboxylation, and borylation process to generate the final products.

**Scheme 29:**
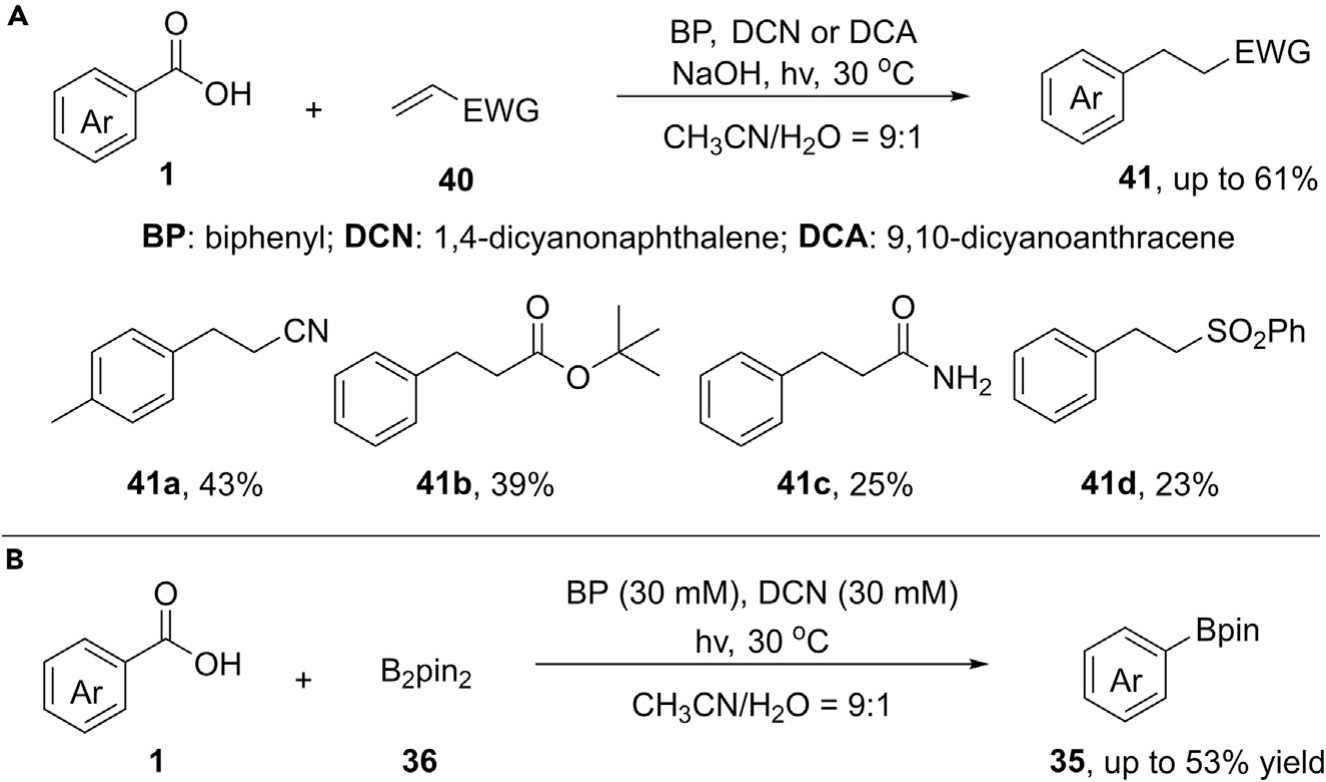
Decarboxylative Radical Addition, Borylation, and Reduction.

Inspired by visible-light-induced decarboxylation processes, the group of Yoshimi recently extended this concept to the intermolecular decarboxylative radical addition reaction between electron-poor alkenes and aryl carboxylic acids under mild conditions (30°C) (Kubosaki et al., 2020) (Scheme 29). As a result, a wide variety of electron-poor alkenes, such as acrylonitrile, acrylamide, acrylate, and phenyl vinyl sulfone, coupled smoothly with benzoic acids upon visible light or UV irradiation. However, the benzoic acids bearing with strong electron-donating groups such as 4-methoxyl and 2,4-dimethoxyl benzoic acids were not tolerated at current stage. Notably, this reaction system can be further applied for the decarboxylative borylation and reduction reactions.

### Acyl Radical Reactions

The reaction of acyl radicals could be traced back to the beginning of last century (Chatgilialoglu et al., 1999; Ryu, 2001). These reactive radicals have been established as important synthetic building blocks for the construction of carbonyl compounds. Not surprisingly, over past decades, considerable efforts have been devoted to this important realm (Zhu et al., 2020; Peng et al., 2019). Typically, acyl radicals are generated from the corresponding aldehydes, acyl chlorides, or some pre-generated precursors such as thioesters, selenoesters, telluroesters, and acylcobalt (III) derivatives under elevated temperature, in the presence of tin reagents or peroxides, or with UV irradiation. The radical carboxylation of alkyl or aryl radical in high pressure of carbon monoxide (CO) is also often used to generate acyl radical intermediates. In addition, the radical decarboxylation of α-keto acids provides an alternative approach to acyl radical species (Liu et al., 2014). Accordingly, Kolbé-type reaction, Minisci reaction, and various radical addition reactions have been developed over past decades. However, the direct generation of acyl radical from benzoic acids is largely unexploited due to the limitation of convenient catalytic systems.

**Scheme 30:**
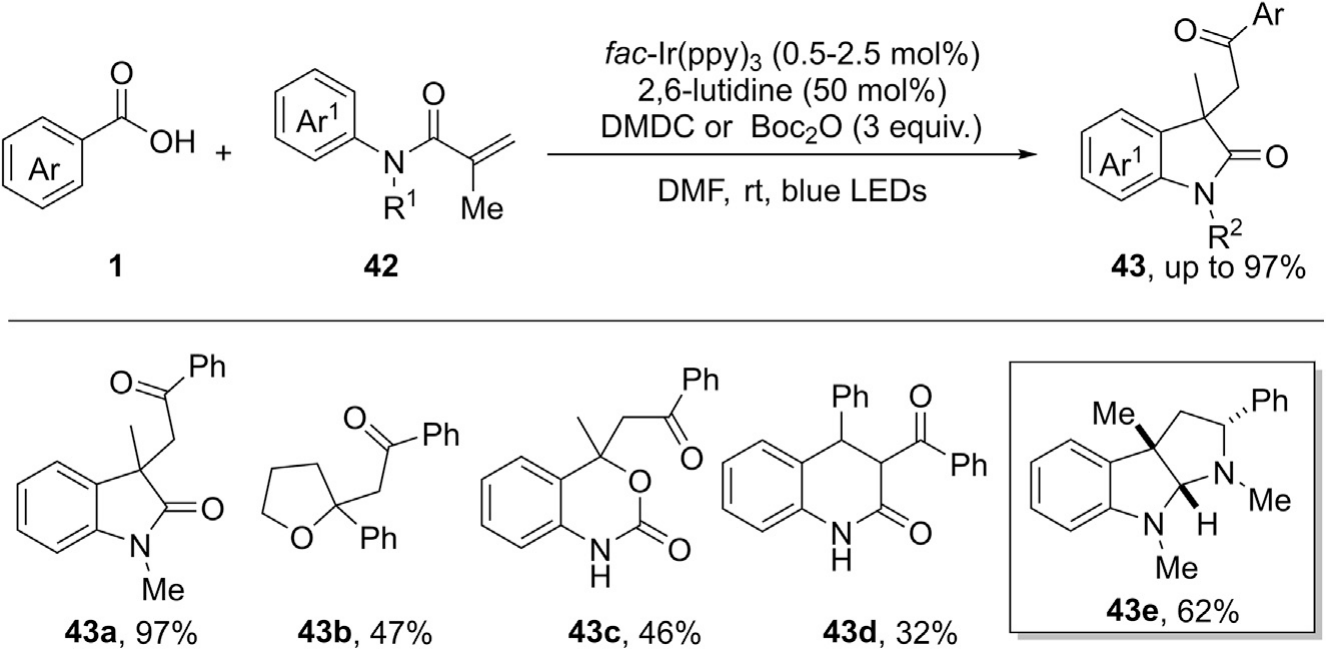
Visible-Light-Induced Radical Acylarylation of Methacrylamides.

An unprecedented work in this field was reported by Wallentin et al., in 2015 (Bergonzini et al., 2015) (Scheme 30). They discovered a novel photocatalytic procedure for the formation of acyl radicals from benzoic acids under mild condition. In this reaction, benzoic acids are firstly activated by dimethyl dicarbonate (DMDC) for *in situ* generation of reactive anhydrides, which could be easily reduced by excited photocatalyst *fac*-∗Ir^III^(ppy)_3_ to give acyl radical species with the extrusion of CO_2_. The methodology has been applied in the radical acylarylation of methacrylamides, delivering 3,3-disubstituted 2-oxindoles in moderate to good yields. However, aliphatic carboxylic acids are not tolerated in this transformation. Notably, the current reaction can be extended to other styrene-type systems for the construction of furan and quinolinone derivatives. The utility of this protocol was further demonstrated by the concise synthesis of hexahydropyrrolo[2,3-b]indole **43e** through two simple steps. Inspired by this work, Wallentin and co-workers then extended this strategy to a tandem radical addition/semipinacol rearrangement of allyl alcohols for the assembly of 1,4-diketones and 1,4-ketoaldehydes (Bergonzini et al., 2016).

**Scheme 31:**
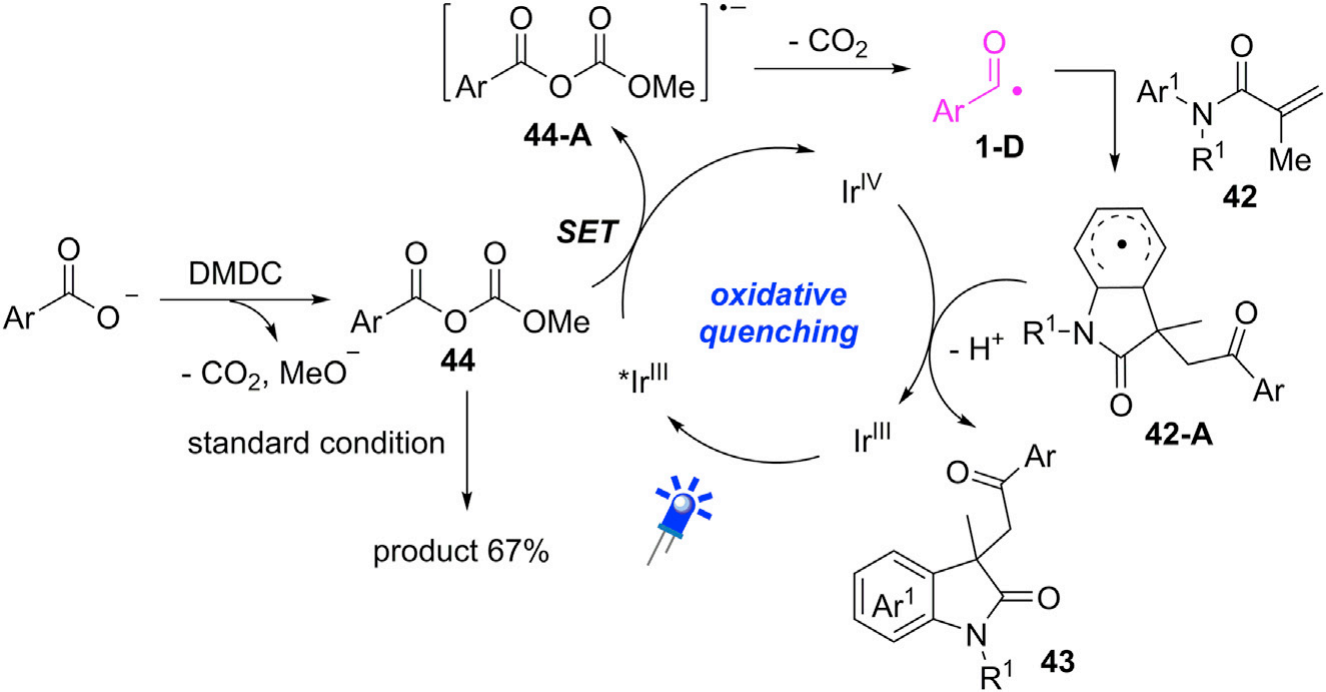
Proposed Mechanism of Light-Induced Radical Acylarylation of Methacrylamides.

The reaction begins with the generation of anhydrides **44** from benzoic acids in the presence of DMDC and 2,6-lutidine (Scheme 31). Subsequently, an SET reduction of **44** by excited photocatalyst ∗Ir^III^ gives the radical anion **44-A**, which proceeds by a decarboxylative process resulting in the formation of acyl radical species **1-D**. Then, the addition of **1-D** to C=C bonds gives rise to a new radical **42-A**. A sequential oxidation and deprotonation of intermediate **42-A** affords the final product and finishes the photocatalytic cycle. When anhydride **44** was subjected to the standard reaction conditions, the reaction proceeded smoothly to give the desired product in 67% yield, which supports the intermediacy of anhydrides in this reaction.

**Scheme 32:**
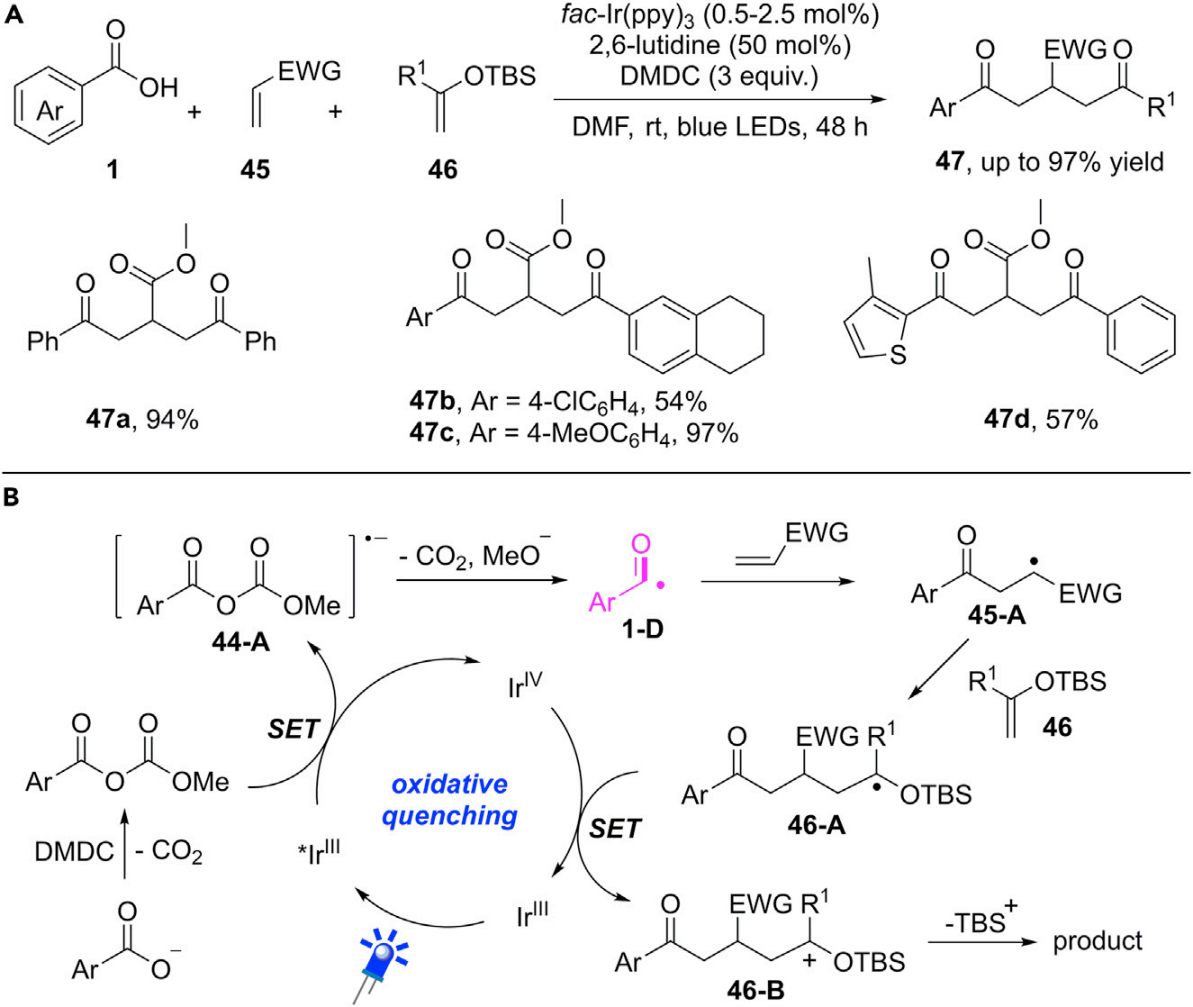
Visible-Light-Induced Multicomponent Reaction of Benzoic Acids.

In continuation of their efforts to acyl radical reactions, Wallentin et al. further developed an interesting multicomponent reaction (MCR) of benzoic acids, methyl acrylates, and silyl enol ethers for the convenient synthesis of δ-diketones (Pettersson et al., 2017) (Scheme 32). Strategically, the electron-rich acyl radical prefers to react with the electron-poor methyl acrylates and then the newly generated electron-poor C−radical tends to couple with electron-rich silylenolethers. In general, benzoic acids bearing an electron-rich group are more efficient than electron-poor substrates. The scalability and synthetic value of this methodology have been evaluated by a gram-scale reaction and the rapid construction of trisubstituted pyridine and cyclopentene from commercially available materials in two steps.

The generation of acyl radical is similar to the previously mentioned process. The benzoic acid is firstly activated by DMDC to give the corresponding anhydride (Scheme 32). Then, the oxidative quenching of photoexcited ∗Ir^III^ by anhydride leads to a radical anion **44-A**, followed by a fragmentation process to generate acyl radical intermediate **1-D**. The selective radical addition of electron-rich acyl radical to an electron-poor alkene forms a new electron-poor radical **45-A**, which prefers to combine with an electron-rich olefin to form C−radical **46-A**. Then, intermediate **46-A** undergoes an SET oxidation/elimination of TBS^+^ cascade, furnishing the final product and regenerating the ground-state of photocatalyst.

**Scheme 33:**
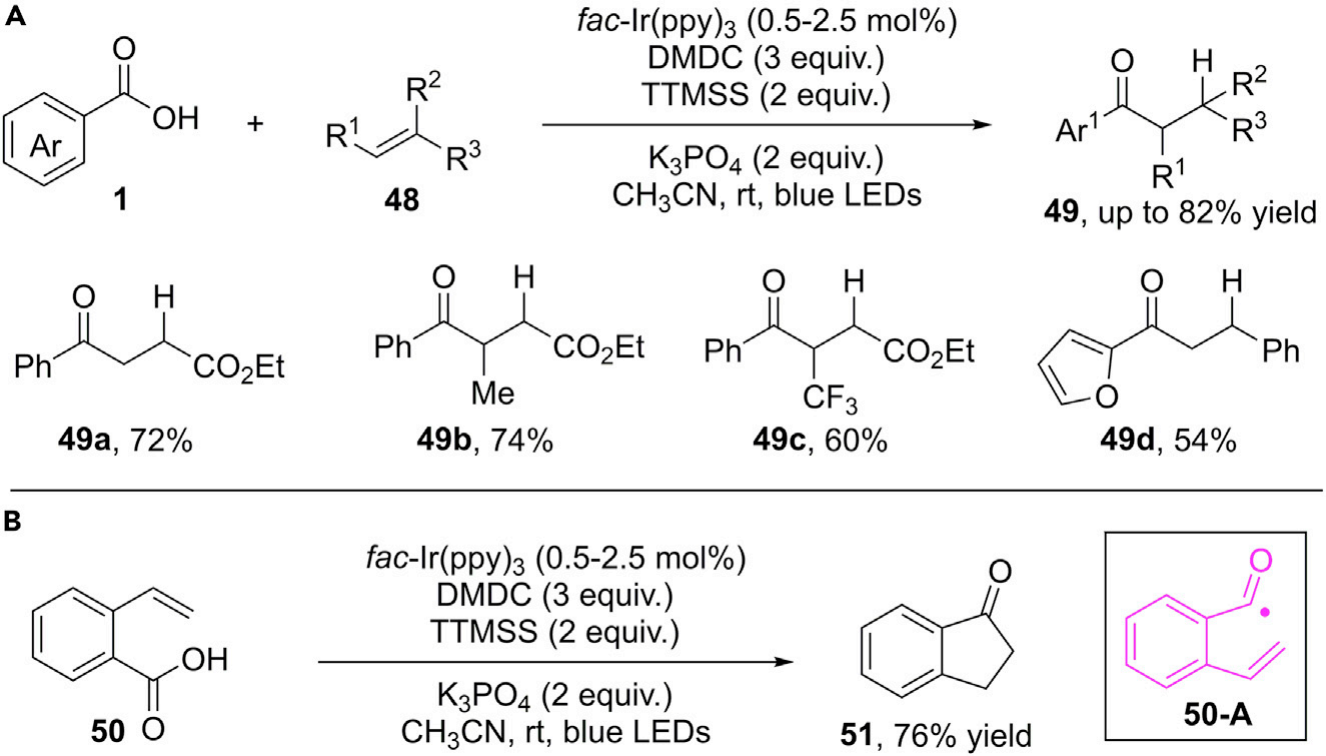
Visible-Light-Induced Photocatalytic Hydroacylation of Alkenes.

Zhu and Yu et al. reported a radical hydroacylation of alkenes by rational combination of a visible-light-induced SET activation and an HAT step (Zhang et al., 2017) (Scheme 33). The same concept has been used to generate active acyl radicals from benzoic acids and tris(trimethylsilyl)silane (TTMSS) acted as an efficient hydrogen source. Compared with transitional metal-catalyzed hydroacylation process, this protocol features mild condition, good functional group tolerance, and broad substrate scope. Both styrenes and aliphatic olefins are compatible in this reaction. Importantly, the intramolecular reaction of 2- vinylbenzoic acid proceeded smoothly, resulting 1-indone in 76% yield. Mechanistic studies validated the photocatalytic SET reduction of anhydrides and H−atom abstraction process of newly generated C−radical from TTMSS in this transformation.

**Scheme 34:**
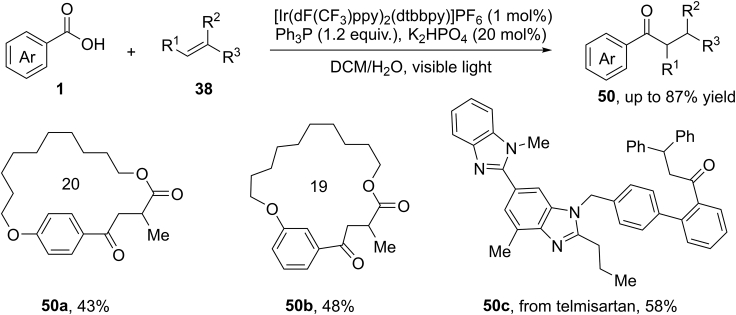
Visible-Light-Induced Deoxygenative Functionalization of Aromatic Carboxylic Acids.

As an extension of this concept, in 2018, Zhu, Xie, and co-workers reported an elegant deoxygenative functionalization of aromatic carboxylic acids for the synthesis of aryl ketones by visible light photocatalysis (Zhang et al., 2018a, 2018b) (Scheme 34). Triphenylphosphine (Ph_3_P) was used as an oxygen transfer reagent. The C−O bond cleavage of carboxylic acids was promoted by a polar/SET crossover between a carboxylate anion and reactive Ph_3_P radical cation intermediate. This reaction has a very broad substrate scope with respect to aromatic acids and alkenes. Both terminal and internal alkenes were effective for this process. However, aliphatic acids and cinnamic acid failed to give the expected products. It is noteworthy that this methodology could be further applied for the construction of cyclophane-braced macrocycloketones, the 3-step rapid synthesis of the drug Zolpidem, and the late-stage modification of biologically important telmisartan, adapalene, and estrone.

**Scheme 35:**
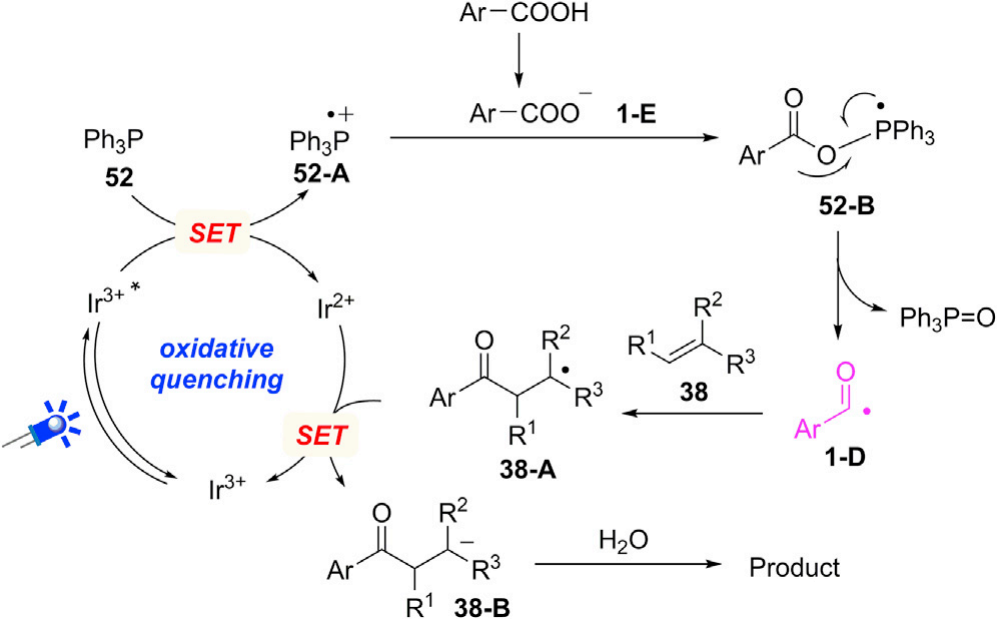
Proposed Mechanism for Deoxygenative Functionalization of Carboxylic Acids

The success of intramolecular hydroacylation reaction suggested the possible intermediacy of acyl radical. In addition, ^18^O-labeling experiments demonstrated that the O−atom of by-product triphenylphosphine oxide originated from carboxylate group rather than water. Based on these results, a plausible mechanism is proposed in Scheme 35; an SET oxidation of Ph_3_P (E_1/2_^red^ = +0.98 V versus SCE) by excited photocatalyst ∗Ir^III^ [E_1/2_^red^ (∗Ir^III^/Ir^II^) = +1.21 V versus SCE] forms triphenylphosphine radical cation **52-A**, which could react with carboxylate anion to give radical intermediate **52-B**. The *β*-C–O bond cleavage of **52-B** results in acyl radical **1-D**, which is subsequently trapped by alkene **38** to give a new C-centered radical **38-A**. Intermediate **38-A** undergoes an SET reduction and protonation sequence to generate the final product.

**Scheme 36:**
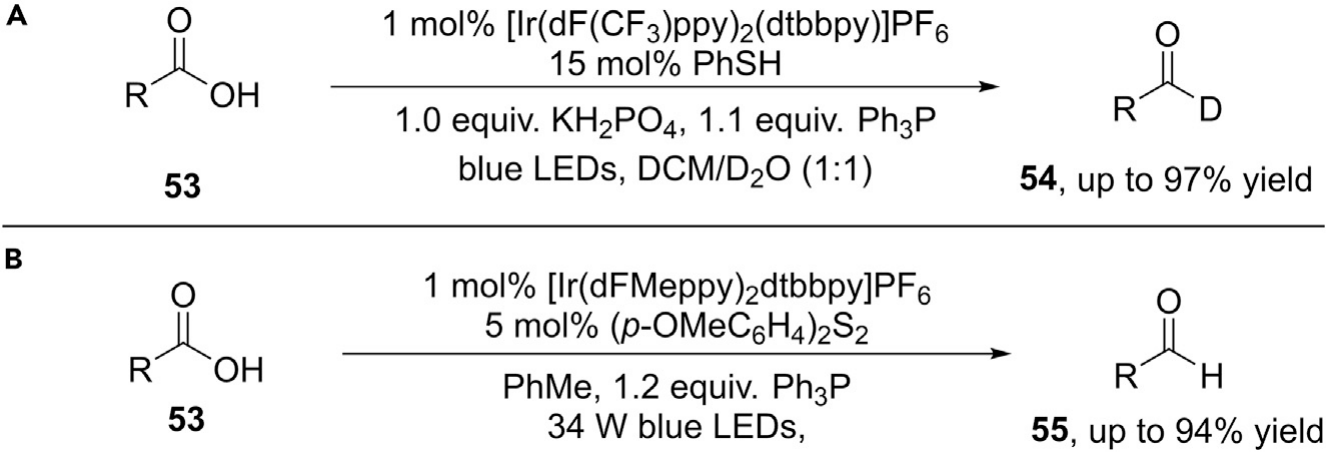
Visible-Light-Induced Deoxygenative Deuteration of Carboxylic Acids.

Shortly thereafter, Xie et al. further developed a facile deoxygenative deuteration of benzoic acids by using the similar strategy (Zhang et al., 2019) (Scheme 36A). Ph_3_P and Ph_2_POEt were used as oxygen transfer reagents for the deoxygenation of aromatic acids and aliphatic acids, respectively. A wide range of aromatic acids and aliphatic acids proved to be suitable for this reaction, furnishing the deuterated aldehydes in generally good yields. The robustness of this transformation is highlighted by the deoxygenative deuteration of some pharmaceuticals and the facile synthesis of D−labeled heterocycles.

Almost at the same time, Doyle and Rovis et al. developed an interesting visible-light-induced deoxygenative reduction of benzylic alcohols and carboxylic acids (Stache et al., 2018) (Scheme 36B). Importantly, the intramolecular cyclizations were also achieved in this reaction, giving ketones and lactones in moderate to good yields. This reaction provides a novel avenue for the activation of strong C−O bonds under mild conditions.

**Scheme 37:**
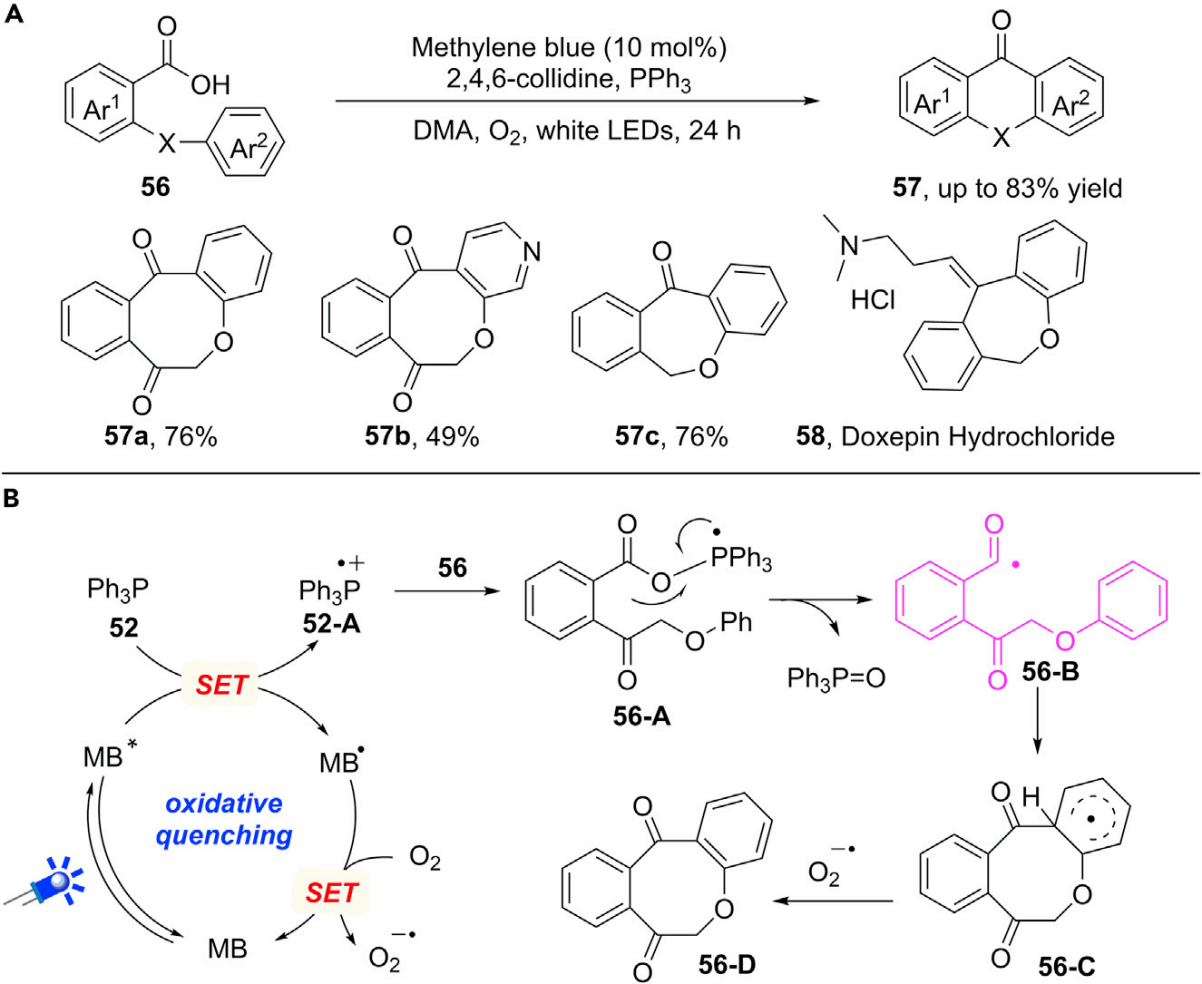
Visible-Light-Induced Intramolecular Acyl Radical Cyclization.

In addition, Chu, Sun, and co-workers applied the concept of intramolecular acyl radical cyclization for the efficient synthesis of dibenzocycloketones under metal-free photocatalytic conditions (Jiang et al., 2019) (Scheme 37A). The inexpensive and commercially available methylene blue (MB) (E_1/2_^ox^ = +1.13 V versus SCE) was employed as an efficient organic photosensitizer in this reaction. Under standard conditions, a diverse set of eight-membered dibenzocycloketones can be obtained in moderate to good yields. Moreover, the substrates bearing a C−linker or heteroatom linkers were also well tolerated in this process, giving the desired products in promising yields. The importance of this reaction was further identified by the synthesis of doxepin hydrochloride for treating chronic pain and depression.

A possible mechanism was outlined in Scheme 37B. Under irradiation by white LEDs, methylene blue arrives to its excited state MB∗. An SET oxidation of PPh_3_ by MB∗ generates Ph_3_P radical cation **52-A** and photocatalyst species MB^⋅^. Then, the SET crossover between a carboxylate anion and reactive Ph_3_P radical cation **52-A** gives the key acyl radical **56-B**, which undergoes an intramolecular cyclization/H−atom abstraction process to give the product. At the same time, the photocatalyst MB^⋅^ is oxidized by atmospheric O_2_ to regenerate the ground state of catalyst MB and then close the catalytic cycle.

### Conclusions

Over the past 10 years, the single electron activation of benzoic acids has been established as a powerful strategy for the transformation of aryl carboxylic acids into high-value compounds. In this context, the active aryl carboxylic radical, aryl radical, and acyl radical species could be efficiently generated from benzoic acids or their NHPI ester and anhydride derivatives by electrocatalysis, visible-light photocatalysis, or in the presence of some external oxidants. Based on these reactive species, a wide range of radical reactions have been developed, such as radical addition, HAT reaction, borylation, and radical cascade reaction. Compared with classic transition metal-catalyzed pathways, these radical reactions generally feature mild condition, broad scope, and good functional group tolerance because of the high efficiency and unique reactivity of radical species. Significantly, this strategy has a promising potential in the construction of some pharmaceutical ingredients and late-stage functionalization of natural products.

Despite these impressive achievements, the following challenges can be found: (1) the development of novel intermolecular radical reactions; (2) the asymmetric radical transformation of aryl carboxylic acids. Because of the high reactivity of radical species, the rational combination of the SET activation strategy with transition metal catalysis (Ni, Cu, and Co) may provide a possibility for the development of intermolecular reactions. In this case, the metal-catalyst could stabilize reactive radical species to *in situ* generated metal-coordinated radicals, which are more stable and perform distinct reactivities. In addition, the merging of mild photoredox catalysis with other chiral catalytic systems such as organo- or transition-metal catalysis would provide a potential solution to the asymmetric radical reactions. We hope this review will provide a handy reference for researchers who are interested in the chemistry of aryl carboxylic acids, stimulating more efforts in this fascinating field.
